# MiR-138 downregulates miRNA processing in HeLa cells by targeting *RMND5A* and decreasing Exportin-5 stability

**DOI:** 10.1093/nar/gkt839

**Published:** 2013-09-19

**Authors:** Jie Li, Ying Chen, Xingliang Qin, Junzhi Wen, Hongmei Ding, Wei Xia, Shaohua Li, Xueting Su, Wei Wang, Hui Li, Qiang Zhao, Tao Fang, Lianghu Qu, Ningsheng Shao

**Affiliations:** ^1^Department of Biochemistry and Molecular Biology, Beijing Institute of Basic Medical Sciences, Beijing 100850, China and ^2^Key Laboratory of Gene Engineering of the Ministry of Education, State Key Laboratory for Biocontrol, Sun Yat-sen University, Guangzhou, 510275, China

## Abstract

MicroRNAs (miRNAs) are a class of non-coding small RNAs that consist of ∼22 nt and are involved in several biological processes by regulating target gene expression. MiR-138 has many biological functions and is often downregulated in cancers. Our results showed that overexpression of miR-138 downregulated target RMND5A (required for meiotic nuclear division 5 homolog A) and reduced Exportin-5 stability, which results in decreased levels of pre-miRNA nuclear export in HeLa cells. We also found that miR-138 could significantly inhibit HeLa cell migration by targeting RMND5A. Our study therefore identifies miR-138–RMND5A–Exportin-5 as a previously unknown miRNA processing regulatory pathway in HeLa cells.

## INTRODUCTION

MicroRNAs (miRNAs) are a class of non-coding small RNAs of ∼22 nt that induce target mRNA degradation or translational repression by complementary base pairing (full or incomplete) to the 3′ untranslated region (3′UTR) (1–4). miRNA expression profiles vary in different tissues, organs and cell types and also at different stages of cell growth and development. miRNA expression is closely related to many physiological and pathological processes, such as cell differentiation, apoptosis, development, lipid metabolism, hormone secretion, tumor formation and viral infection (5–17). In the nucleus, the miRNA-coding sequence is first transcribed to a primary miRNA that is cleaved by the RNase Ⅲ Drosha–DGCR8 (18–23). Exportin-5, a Ran-GTP-dependent transport protein, mediates the translocation of the miRNA precursor from the nucleus to the cytoplasm (24–27). Cytoplasmic pre-miRNA is subsequently processed by a second RNase Ⅲ, Dicer, to liberate mature miRNA (28,29). This then associates with Argonaute proteins to form the RNA-induced silencing complex that binds to the 3′UTR of target mRNAs to perform its biological functions (30,31).

The human miR-138 family consists of hsa-miR-138-1 and hsa-miR-138-2 located on chromosomes 3p21.32 and 16q13, respectively (32–34). MiR-138 has various biological functions, including roles in tumor progression and metastasis, cell differentiation, DNA damage and disease. Liu *et al.* (35) reported that head and neck squamous cell carcinomas (HNSCCs) exhibiting the most mesenchymal-like features had the lowest levels of miR-138 expression. MiR-138 inhibits HNSCC cell invasion and induces cell cycle arrest and apoptosis. Furthermore, miR-138 targets EZH2, VIM and ZEB2, thereby downregulating expression of the downstream E-cadherin gene (*CDH1*) and affecting epithelial–mesenchymal transition (36). In tongue squamous cell carcinoma (TSCC) cells, miR-138 inhibits migration and invasion by targeting *RHOC* and *ROCK2*, which belong to the Rho GTPase signaling family (37). MiR-138 also suppresses ovarian cancer cell invasion and metastasis by targeting SOX4 and HIF-1α (38). miR-138 has also been reported to target *GANI2* (a cancer-promoting factor), to inhibit TSCC cell proliferation, induce cell cycle arrest and promote apoptosis (39). Similarly, miR-138 targeting of *FOSL1* (which encodes Fos-like antigen 1) reduces the expression of the downstream gene, *Snai2*, which inhibits E-cadherin expression in squamous cell carcinoma (SCC) cells (40). Regarding its role in cell differentiation, miR-138 dynamically regulates neural development by controlling the shape and size of dendrites and thereby influences long-term memory (41). MiR-138 is significantly downregulated during adipogenic differentiation, whereas the overexpression of miR-138 inhibits adenovirus early region 1A-like inhibitor of differentiation 1 and thus reduces lipid droplet accumulation (42). In esophageal SCC, downregulation of miR-138 sustains NF-κB activation and promotes lipid raft formation (43). MiR-138 targets focal adhesion kinase to inhibit osteoblast differentiation, and its expression is reduced during osteogenic differentiation (44). Furthermore, miR-138 targets the histone, H2AX, and thus miR-138 overexpression inhibits homologous recombination of chromosomes and enhances cell sensitivity to DNA-damaging agents such as cisplatin, camptothecin and ionizing radiation (45). In addition, miR-138 downregulation is associated with the development of thyroid carcinoma and multidrug resistance in leukemia cells (46,47). MiR-138 is becoming a hot topic of study, as it has so many different functions and is usually downregulated during pathological processes, especially during carcinogenesis (35,39,48,49). HeLa cells contain low levels of mature miR-138 (50) and thus provide a good model system for studying the functional effects of miR-138 overexpression. In this study, the overexpression of miR-138 in HeLa cells specifically targeted *RMND5A*, resulting in the inhibition of HeLa cell migration. Exportin-5 stability was also affected, thereby regulating the downstream expression of several miRNAs.

## MATERIALS AND METHODS

### Cell culture and transfection

MiR-138 mimic and anti-miR-138 (miR-138 inhibitor, 2′-O-methyl modification) were synthesized by RiboBio. SiRNAs were synthesized by GenePharma. HeLa cells were grown in Dulbecco’s modified Eagle’s medium (GIBCO-Invitrogen) supplemented with 10% bovine growth serum (HyClone). Cells were seeded into plates 24 h before transfection. Transfection experiments were performed using 20 nM siRNA, 20 nM miRNA mimics or 50 nM anti-miR and Lipofectamine 2000 (Invitrogen), according to the manufacturer’s instructions. A random RNA duplex (GenePharma) was used as a negative control. When required, 24 h after transfection, the proteasome inhibitor MG132 (SelleckChemicals) was added at a concentration of 20 μM for 6 h. The miRNA mimics and siRNAs were as follows:

miR-138 mimic, 5′-AGCUGGUGUUGUGAAUCAGGCCG-3′ (sense); Control mimic, 5′-CUCCGAACGUGUCACGU-3′ (sense); RMND5A siRNA, 5′-CACCAUAUGUUCACCUACU-3′ (sense); control siRNA, 5′-UUCUCCGAACGUGUCACGU-3′ (sense); Dicer siRNA, 5′-UGCUUGAAGCAGCUCUGGA-3′ (sense); Exportin-5 siRNA, 5′-UGUGAGGAGGCAUGCUUGU-3′ (sense); RanBPM siRNA, 5′-GGCCACACAAUGUCUAGGA-3′ (sense); Ran siRNA, 5′-GAAAUUCGGUGGACUGAGAUU-3′ (sense).

### Plasmid construction

*RMND5A* cDNA (pCMV-RMND5A) was obtained from OriGene (USA). cDNAs encoding full-length open reading frames and deletion mutants of *RMND5A* were obtained by PCR using pCMV-RMND5A, Pfu DNA polymerase and synthetic oligonucleotide primers incorporating restriction sites. PCR products were ligated into the pcDNA6/myc-His B vector according to the manufacturer’s protocol (Invitrogen) and then sequenced to confirm the absence of mutations. pCMV-Dicer1, pCMV-RanBPM and pCMV-Exportin-5 were also obtained from OriGene. For *in vitro* translation, RMND5A, RanBPM and Exportin-5 cDNA were separately cloned into the pF3K WG (BYDV) Flexi® vector according to the manufacturer’s protocol (Promega). Primer sequences were as follows (sequence from 5′ to 3′): RMND5A Full F, CCC***AAGCTT***GCCACCATGGATCAGTGCGTGACGGTG (*Hind*Ⅲ); RMND5A Full R, CCG***CTCGAG***CGGAAAAATATCTGTTTGGCAT (*Xho*I); RMND5AΔLisH R1, CTTCTGACTTGGGTCGTCTGCCTGCCAGCA; RMND5AΔLisH F2, TGCTGGCAGGCAGACGACCCAAGTCAGAAG;RMND5AΔCTLH R1, TAATGCCTCTCGCTGTTCCTTCTGACTTGG; RMND5AΔCTLH F2, CCAAGTCAGAAGGAACAGCGAGAGGCATTA; RMND5AΔCRA R1, CTGCCTCTGTTCAATCTGATTTGTGGTTCC; RMND5AΔCRA F2, GGAACCACAAATCAGATTGAACAGAGGCAG; RMND5AΔRING R, CCG***CTCGAG***CGTATAGAGTGATACCAGCACT (*Hind*Ⅲ)； pF3K-RMND5A F, GCGATCGC***GCCACC***ATGGATCAGTGCGTGACGGTG (*Sgf*I)；pF3K-RMND5A R, AGCTTT***GTTTAAAC***ATCTGTTTGGCATCTCCTG (*Pme*I); pF3K-Exportin-5 F, GCGATCGC***GCCACC***ATGGCGATGGATCAAGT (*Sgf*I); pF3K-Exportin-5 R, AGCTTT***GTTTAAAC***TCAGGGTTCAAAGATGGT (*Pme*I); pF3K-RanBPM F, GCGATCGC***GCCACC***ATGTCCGGGCAGCCGCCG (*Sgf*I); pF3K-RanBPM R, AGCTTT***GTTTAAAC***CTAAGCGGTATGCATATT (*Pme*I).

### Quantitative real-time PCR

Relative mRNA levels were determined by qRT-PCR using a Stratagene MX3000P system, SYBR Premix Ex Taq (TaKaRa) and specific primers. CT values were determined using MX3000p software (v4.10) with amplification-based threshold determination and adaptive baseline analysis options. Glyceraldehyde 3-phosphate dehydrogenase (GAPDH) was used as an endogenous control. Results represent at least three experiments. Primer sequences were as follows:

RMND5A F, 5′-GTACCTGAGACAAGGGATT-3′; RMND5A R, 5′-AACCTGCTGAGAAACTG-3′; RHOC F, 5′-ACCTGCCTCCTCATCGTCTTC-3′; RHOC R, 5′-CACCTGCTTGCCGTCCAC-3′; ROCK2 F: 5′-TGCCTTCATCTGTAGACCTCTG-3′; ROCK2 R, 5′-CCATCAACGTGGAGAGCTTG-3′; Dicer F: 5′-TGCCAGTTGGGAAAGAGACTGTTAA-3′; Dicer R: 5′-TATGGGTTTGGCCGTCAGTATT-3′; XPO5 F, 5′-CACAGACTGCTGATGGAGGA-3′; XPO5 R: 5′-TGACTTGGATGGGTTGTGAA-3′; GAPDH F: 5′-GAAGGTGAAGGTCGGAGTC-3′; GAPDH R, 5′-GAAGATGGTGATGGGATTTC-3′.

miRNAs and pre-miRNAs were quantified using the All-in-One miRNA qRT-PCR Detection Kit (GeneCopoeia, Inc.). HeLa cell nuclear and cytoplasmic RNAs were obtained using Cytoplasmic and Nuclear RNA Purification Kits (Norgen). For single-step cDNA synthesis, poly(A) polymerase was used to add poly(A) tails to the 3′ end of miRNAs, and Moloney Murine Leukemia Virus reverse transcriptase and a unique Oligo(dT) adaptor primer were used to reverse transcribe poly(A) miRNAs. An All-in-One qPCR Mix containing SYBR Green was used to detect specific reverse-transcribed miRNAs using a Universal Adaptor PCR primer and a miRNA-specific primer. Cytoplasmic RNAs were normalized to GAPDH mRNA, and nuclear RNAs were normalized to U6 small RNA. Results are expressed in arbitrary units and are representative of three independent experiments. Primer sequences were as follows:

Oligo(dT) adaptor primer R, 5′-GCGAGCACAGAATTAATACGACTCACTATAGGTTTTTTTTTTTTTTTTTTVN-3′; Universal Adaptor primer, 5′-GCGAGCACAGAATTAATACGAC-3′; U6 F, 5′-CTCGCTTCGGCAGCACA-3′; pre-miR-105–1 F, 5′-TGTGCATCGTGGTCAAATGCTC-3′; pre-miR-19a F, 5′-GCAGTCCTCTGTTAGTTTTGC-3′; pre-miR-7–1 F, 5′-TTGGATGTTGGCCTAGTTCTGTG-3′; pre-miR-128–1 F, 5′-TGAGCTGTTGGATTCGG-3′; pre-miR-93 F, 5′-CTGGGGGCTCCAAAGTGCTGT-3′; pre-miR-30a F, 5′-GCGACTGTAAACATCCTCGAC-3′; pre-miR-17 F, 5′-GTCAGAATAATGTCAAAGTGCT-3′; pre-miR-107 F, 5′-CTCTCTGCTTTCAGCTTCTT-3′; pre-miR-128–1 F, 5′-TGTGCAGTGGGAAGGGGGGCCGATACA-3′; pre-miR-124–3 F, 5′-TGAGGGCCCCTCTGCGTGTT-3′; pre-miR-142 F, 5′-GACAGTGCAGTCACCCATAAA-3′; miR-128 F, 5′-GCCGCTCACAGTGAACCGGTCT-3′; miR-7 F, 5’-CGGCGGTGGAAGACTAGTGATT-3′; miR-93 F, 5′-CTGGGGGCTCCAAAGTGCTGTT-3′; miR-30a F, 5′-GCGACTGTAAACATCCTCGA-3′; miR-17 F, 5′-CGGCGCAAAGTGCTTACAGTG-3′; miR-107 F, 5′-GCCGCAGCAGCATTGTACAGG-3′; miR-23a F, 5′-GCGTGATCACATTGCCAGGGAG-3′; miR-142 F, 5′-GACAGTGCAGTCACCCATAAA-3′; miR-124 F, 5′-TCGGCGTGTTCACAGCGGACCTT-3′.

### Immunoprecipitation, identification of protein complex members and western blot analysis

HeLa cells were grown in 6-well plates and transfected with the indicated plasmids and miRNA mimic or siRNA using Lipofectamine 2000 (Invitrogen). Cells were washed with phosphate-buffered saline and lysed for 20 min in cold lysis buffer [50 mM Tris–HCl (pH 7.4); 1% NP-40; 0.25% sodium deoxycholate; 150 mM NaCl; 1 mM each EDTA, PMSF, Na_3_V0_4_, and NaF; 1 μg/ml each aprotinin, leupeptin, and pepstatin]. Extracts were clarified by centrifugation (10 000*g*) for 20 min at 4°C, and then 500 μg of protein was incubated with 2 μg of Myc-tag antibody or immunoglobulin G (IgG) for 4 h at 4°C with agitation. A total of 50 μl of protein A/G magnetic beads (Millipore) was added to each sample and incubated for 1 h. After washing three times with lysis buffer, complex components were separated using 10% Sodium dodecyl sulfate (SDS)–PAGE. Electrophoresis was carried out at a constant voltage of 50 V using 3-(N-morpholino)propanesulfonic acid SDS running buffer (Invitrogen) for ∼25 min. The proteins were visualized with Coomassie blue. Entire gels were diced into small pieces (1–2 mm). The proteins were destained, excised and digested as described previously (51) before NanoLC-HDMS MS/MS analysis and a Mascot database search. For western blot analysis, samples were separated using 10% SDS–PAGE and transferred to nitrocellulose membranes. Membranes were blocked in Tris-buffered saline (TBS) containing 5% non-fat dry milk and 0.05% Tween-20 (TBST-MILK) for 30 min at 25°C with shaking, and then incubated with primary antibody (following the manufacturer’s instructions) for 2 h at 25°C. Samples were then washed with TBST (TBS-0.05% Tween-20) and incubated for 1 h with horseradish peroxidase-conjugated secondary antibody (1:5000 dilution). Protein bands were visualized using enhanced chemiluminescent substrate, according to the manufacturer’s protocol (Thermo). For immunoblots, primary antibodies used were anti-β-Actin, anti-ROCK2, anti-Exportin-5, anti-RMND5A and anti-RanBPM from Santa Cruz Biotechnology, anti-Myc-tag from Abnova, anti-HA-tag from Sungene Biotech and anti-RhoC from Cell Signaling.

### Immunofluorescence microscopy

Twenty-four hours after transfection treatment, cells were ﬁxed with 4% paraformaldehyde/PBS for 20 min. Samples were preblocked with 1% Donkey serum/PBS and incubated respectively overnight with the following primary antibodies: anti-RMND5A (Host: Goat), anti-Exportin-5 (Host: Rabbit) and anti-RanBPM (Host: Goat) from Santa Cruz Biotechnology. After washing, cells were incubated with secondary antibodies: Donkey anti-Goat-Cy3 and Donkey anti-rabbit-FITC from Cwbiotech at room temperature. Nuclei were counterstained with 4′, 6-diamidino-2-phenylindole (DAPI). Image acquisition was done with Olympus FV1000 confocal microscope.

### *In vitro* translation and protein–protein interaction assays

*In vitro* translation was performed with a TnT® sp6 High-Yield Wheat Germ Protein Express System (Promega). Each 50 μl of reaction mixture contained a total of 3 μg of plasmid DNA and were incubated at 25°C for 2.5 h. One-tenth of each *in vitro* translation reaction was set aside to identify the translated proteins, and the volume of each lysate was increased to 150 μl by adding buffer [50 mM HEPES (pH 7.2), 10 mM NaPO_4_ (pH 7.0), 250 mM NaCl, 0.2% NP-40, 0.1% Triton X-100, 0.005% SDS and 2.5 mM β-mercaptoethanol] supplemented with protease inhibitor cocktail. Immunoprecipitation and western blot analyses were performed as described earlier in the text.

### Ubiquitination assays

In ubiquitination assays, 20 μM MG132 was added into cell cultures 8 h before cells harvesting. Exportin-5 was transfected into HeLa cells along with HA–ubiquitin Exportin-5 was isolated by immunoprecipitation under denaturing condition (52) to inactivate deubiquitinating enzymes and disrupt protein complexes. For transfection models, following Exportin-5 denaturing immunoprecipitation, the Exportin-5–ubiquitin conjugates were detected by immunoblotting using anti-HA-tag.

### Dual luciferase reporter assay

Three fragments of the *RMND5A* 3′UTR, two containing a single miR-138 conservative binding site (33–319 or 640–935) and one containing both miR-138 binding sites (1–3760), were cloned into the *Xba*I and *Nde*I sites of the pGL3-Control Vector (Promega) and named pGL3-R-1, pGL3-R-2 and pGL3-F, respectively. MiR-138 target binding sites were mutated by deletion the putative binding sequence using the MutanBEST mutation kit (TaKaRa) and cloned into the pGL3-Control vector. Mutant constructs corresponding to pGL3-R-1 and pGL3-R-2 were named pGL3-R-1 mu and pGL3-R-2 mu, respectively. Four fragments of the *DICER1* 3′UTR were also cloned into the *EcoR*I and *Xba*I or *Xba*I and *Nde*I sites of the pGL3-Control Vector and named pGL3-D-1, pGL3-D-2, pGL3-D-3 and pGL3-D-4, respectively. The full-length *XPO5* 3′UTR was also cloned into the *EcoR*I and *Nde*I sites of the pGL3-Control Vector and named pGL3-E. pGL3 vectors, the pRL-CMV vector (used as an internal control for transfection efficiency) and miR-138 mimic or control siRNA were co-transfected into HeLa cells using Lipofectamine 2000 (Invitrogen), according to the manufacturer’s instructions. After 48 h, reporter activity was measured using a dual luciferase reporter gene assay kit (Promega). Primers for cloning (bold italic for restriction sites) were as follows:

RMND5A F, 5′-TGC***TCTAGA***AGAGATAACTTTAGTTTG-3′; RMND5A R, 5′-GGAATTC***CATATG***CCCACTACAGCTTGAAACT-3′; RMND5A-1 F, 5′-GC***TCTAGA***AACTGAATCGTGGGTGCATT-3′; RMND5A-1 R, 5′-GGAATTC***CATATG***GGGAGGTCAATTTCCGTTTC-3′; RMND5A-2 F, 5′-GC***TCTAGA***AACTGGCTGGTGTGAGAAGTC-3′; RMND5A-2 R, 5′-GGAATTC***CATATG***CTGGAACATGCTGCTTTCCT-3′; RMND5A-1mu F, 5′-GTTGTCATTCAATGCAGGTT-3′; RMND5A-1mu R, 5′-TATTTACTTGATCAGAAATG-3′; RMND5A-2mu F, 5′–GATGTCAAACATTGTGTATC-3′; RMND5A-2mu R, 5′–AAGACACATATACAATACAC-3′; Dicer-1 F, GC***GAATTC***ATCGGAAAGGAAGACTTAAAGTT; Dicer-1 R, G***TCTAGA***CCAAAGATGGAAATAATGAGCTA; Dicer-2 F, GC***GAATTC***AAACTGCCGTAATTTTGATACAT; Dicer-2 R, G***TCTAGA***TTTTAACTCAGTAACCAGGGGAT; Dicer-3 F, GC***GAATTC***ACAGTTTGAAGCATTCTGTGATCCACCAGCA; Dicer-3 R, G***TCTAGA***TGAAAAGTAATCAGTGGTTGGAA; Dicer-4 F, GC***TCTAGA***TTCCAACCACTGA; Dicer-4 R, G***CATATG****CATATG*TTATTGGAGATTTACTTGGCTAC; XPO5 F, G***GAATTC***ATCAAGCTTTTGGGCA; XPO5 R: GGAATTC***CATATG***CTGTACGAAACTGAGAT.

### Monolayer wound healing assay

HeLa cells were transfected with miR-138 mimic, RMND5A siRNA or control siRNA. Cells in duplicate wells of a 24-well plate were transfected using Lipofectamine 2000. After 24 h, cells in duplicate wells were combined and added to a single well of a 6-well culture dish. Before seeding the cells, two parallel lines were drawn on the underside of each well with a marker pen. After cells had become adherent, two (or more) parallel scratches, or ‘wounds’, ∼400 μm wide were made perpendicular to the marked lines using a P1000 pipette tip (Fisher). The migration of cells into the ‘wounds’ was observed using an inverted microscope (IX71, Olympus), and images of areas flanking the intersections of the ‘wound’ and the marked lines were taken at regular intervals over the course of 12–36 h.

### Transwell migration assay

HeLa cells were treated with miR-138 mimic, RMND5A siRNA or control siRNA and Transwell migration assays were performed in 6.5-mm Transwell chambers with 8-μm pores (Corning Costar, Corning, NY, USA). The underside of each membrane was coated with 20% fetal bovine serum for 2 h. Approximately 1 × 10^4^ cells were seeded into the upper chambers in 100 μl of serum-free medium; lower chambers contained 500 μl of medium with 10% serum. Treatments were added to both chambers. After cells were allowed to migrate, the medium in the upper chamber was aspirated, and cells on the upper side were removed with a cotton swab. Cells on the lower side of the membrane were stained by DAPI. Images were captured with IX71 fluorescence microscope (Olympus, Japan). For each experiment, the number of cells in nine random ﬁelds on the underside of the ﬁlter was counted, and three independent ﬁlters were analyzed.

### The 3-(4,5-dimethylthiazol-2-yl)-2,5-diphenyltetrazolium bromide cell viability assay

HeLa cells (1 × 10^4^) were seeded in 100 μl of medium per well into 96-well plates and incubated (37°C, 5% CO_2_) overnight. After treatment, 10 μl of 3-(4,5-dimethylthiazol-2-yl)-2,5-diphenyltetrazolium bromide (MTT) solution (5 mg/ml) was added to each well and incubated for 4 h. Following this, culture medium was removed, wells were dried and formazan was resolved with 100 μl/well dimethyl sulfoxide for 30 min. The optical density was measured at 560 nm, and the background absorbance at 650 nm was subtracted.

### Microarray assay

mRNA and miRNA microarray assays were performed by a service provider (LC Sciences). For the miRNA microarray assay, a sample of total cellular RNA (2–5 µg) was size fractionated using a YM-100 Microcon centrifugal filter (Millipore) and isolated small RNAs (<300 nt) were 3′-extended with a poly(A) tail using poly(A) polymerase (New England Biolabs). An oligonucleotide tag was ligated to the poly(A) tail for fluorescent staining, and different fluorescent tags were used for each of two RNA samples in dual-sample experiments. Hybridization was performed overnight on a Paraflo microfluidic chip using a microcirculation pump (Atactic Technologies). After hybridization, fluorescence labeling was detected using tag-specific Cy3 and Cy5 dyes. Hybridization images were collected using a laser scanner (GenePix 4000B, Molecular Devices) and digitized using Array-Pro image analysis software (Media Cybernetics). For two-color experiments, the ratio of the two sets of signals (log_2_ transformed and balanced) and the *P*-values of the *t-*test were calculated. Differential detection of mRNA signals was defined as a fold difference (log_2_ ratio) <−1.00 (downregulated) or >1.00 (upregulated) with *P* < 0.05. Differential detection of miRNA signals was defined as a fold difference (log2 ratio) <−0.3 (downregulated) or >0.3 (upregulated) with *P* < 0.1.

### Protein stability assay

HeLa cells were transfected with miR-138 mimic, RMND5A siRNA or control siRNA for 24 h, and then transfected with an Exportin-5 expression vector. After a further 24 h, cycloheximide (100 µg/ml) was added to the cells, and cells were harvested after 0, 2, 4 or 8 h. Protein concentrations were determined by the Bradford method, and protein levels were analyzed by western blotting, as described earlier in the text. Band intensities were measured by densitometry using ImageJ software (rsb.info.nih.gov/ij), and Exportin-5 levels were normalized to those of β-actin. The rate of Exportin-5 degradation was determined in three independent experiments.

### Statistical analysis

The data were expressed as means ± s.d. Differences were assessed by two tailed Student’s *t*-test using the Excel software. *P* < 0.05 was considered to be statistically signiﬁcant.

## RESULTS

### MiR-138 targets RMND5A in HeLa cells

We used TargetScan5.2 bioinformatics software (http://www.targetscan.org/) to predict that 388 conserved genes in the human genome were miR-138 targets. The set of highest-ranking predicted miR-138 target genes is listed in Table 1. *RMND5A* was the highest-ranking predicted miR-138 target gene, with a score of −1.02. *RMND5A* was also predicted to be a miR-138 target by other commonly used miRNA target gene prediction programs.

**Table 1.** MiR-138 targets identified by bioinformatics prediction and microarray analysis

MiR-138 targets predicted by bioinformatic tools^a^							Transcripts regulated by miR-138 treatment (microarray analysis)^b^	
Gene name^c^	PicTar 4-way	PicTar 5-way	TargetScanS	TargetScan Human 5.2	miRanda (microrna.org)	miRanda (miRBase)	Fold diff.(log2 Ratio of miR-138/control)	*P*-value
RMND5A	1	1		1	1		−1.581873	0.002618
RHOC			1	1	1	1	−0.173956	0.034971
ROCK2	1	1	1	1		1	0.126878	0.07682
DVL2	1		1		1	1	−0.771314	0.079133
PDE3A	1	1	1	1	1		−0.104532	0.042333
GNAI2	1		1	1	1	1	0.141667	0.08064
KIAA1853	1		1	1		1	0.094643	0.04891
JMJD1C	1	1	1	1	1		−0.186473	0.074712
EZH2	1	1	1	1	1	1	−0.248526	0.056931
SLC6A8			1	1	1	1	0.509586	0.403917

^a^Genes that were predicted to be candidate targets of miR-138 were identified by 1 s, and only the top 10 highest-ranking predicted miR-138 target genes are listed.

^b^A complete gene list of the 107 transcripts downregulated by miR-138 treatment is presented in Supplementary Table S1.

^c^Gene names in bold font were predicted to be miR-138 targets by microarray analysis. Fold difference (log_2_ ratio of miR-138/control) < −1.00 and *P* < 0.05.

**Figure 1.**
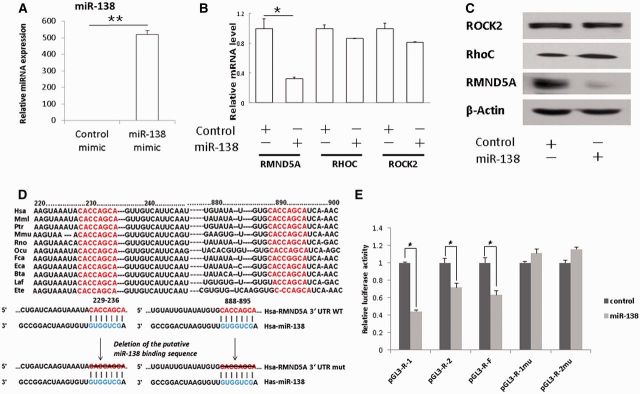
*RMND5A* is a miR-138 target. (**A**) HeLa cells were transfected with miR-138 mimic, and the increased miR-138 level was confirmed by quantitative RT-PCR (qRT-PCR) assays. U6 served as an internal control. (**B**) qRT-PCR assays were performed to examine the effects of miR-138 mimic transfection on endogenous *RMND5A*, *RHOC* and *ROCK2* gene transcription. GAPDH served as an internal control. (**C**) Western blot analyses were performed to examine the effects of miR-138 overexpression on endogenous RMND5A, RhoC and ROCK2 protein levels. (**D**) The predicted highly conserved miR-138 targeting sequence located in the 3′UTR of RMND5A mRNA. (**E**) Dual luciferase reporter assays were performed to test the interaction of miR-138 with wild-type predicted RMND5A 3′UTR targeting sequences (pGL3-R1, pGL3-R2 and pGL3-R) and mutated targeting sequences (pGL3-R1mu, pGL3-R2mu). *P < 0.05. Data are representative of at least three independent experiments (means ± s.d.).

miRNA mimics are small chemically modified double-stranded RNAs that mimic endogenous miRNAs and enable the functional analysis of miRNA by upregulating miRNA activity. As shown in Figure 1A, transfection of the miR-138 mimic into HeLa cells led to an increase in miR-138 levels. We performed microarray-based mRNA differential expression analysis on HeLa cells transfected with a miR-138 mimic and a negative control. Microarray results revealed that levels of 267 transcripts were significantly altered by miR-138 [fold difference (log_2_ ratio of miR-138/control) >1.00; *P* < 0.05]. These included 160 upregulated and 107 downregulated transcripts (Supplementary Table S1). However, *RMND5A* was the only one of the top 10 ranking predicted miR-138 target genes to reach the statistical cut-off [log_2_ (ratio of miR-138/control) <1.00; *P* < 0.05; Table 1].

*RHOC* and *ROCK2* were previously experimentally confirmed as miR-138 targets in TSCC cells (37). However, microarray analysis did not show significant alterations in expression of the two genes by miR-138. As measured by quantitative RT-PCR (qRT-PCR), we observed that miR-138 overexpression significantly downregulated only *RMND5A* mRNA levels in HeLa cells, and that *RHOC* and *ROCK2* mRNA levels were unaffected (Figure 1B). We analyzed the levels of RMND5A, RhoC and ROCK2 proteins in HeLa cells transfected with miRNA mimics, and the results showed a correlation with mRNA levels (Figure 1C), whereby only RMND5A protein expression was significantly reduced. Therefore, miR-138 specifically targets *RMND5A*, thus reducing levels of both mRNA and protein. RhoC and ROCK2 are not functional miR-138 target genes in HeLa cells.

According to bioinformatics analysis, the *RMND5A* 3′UTR is 4630 bp long (NM_022780) and contains two conserved miR-138 target seed sites (at 229–236 and 888–895; Figure 1D) and two non-conserved sites (at 1487–1493 and 1629–1635).To confirm that miR-138 directly targets the *RMND5A* 3′UTR, two wild-type *RMND5A* miR-138 target sites were cloned into the 3′UTR of a luciferase reporter gene, respectively, to construct plasmids pGL3-R-1 and pGL3-R-2. Similar plasmids containing the corresponding mutated target sites, pGL3-R-1 mu and pGL3-R-2 mu, were also constructed. Almost the full length of RMND5A 3′UTR (1–3760 bp) was cloned into the sites of pGL3-Control Vector and named pGL3-R-F. Reporter plasmids and miRNA mimics were co-transfected into HeLa cells. After 48 h, luciferase activity in cells co-transfected with miR-138 mimic was significantly lower than in cells co-transfected with control mimic. However, co-transfection of miR-138 with *RMND5A* mutant target sites fused to a luciferase vector did not suppress luciferase activity compared with controls (Figure 1E). These results indicated that miR-138 directly targets both conserved seed sites in the *RMND5A* 3′UTR.

### RMND5A interacts with Exportin-5

**Figure 2.**
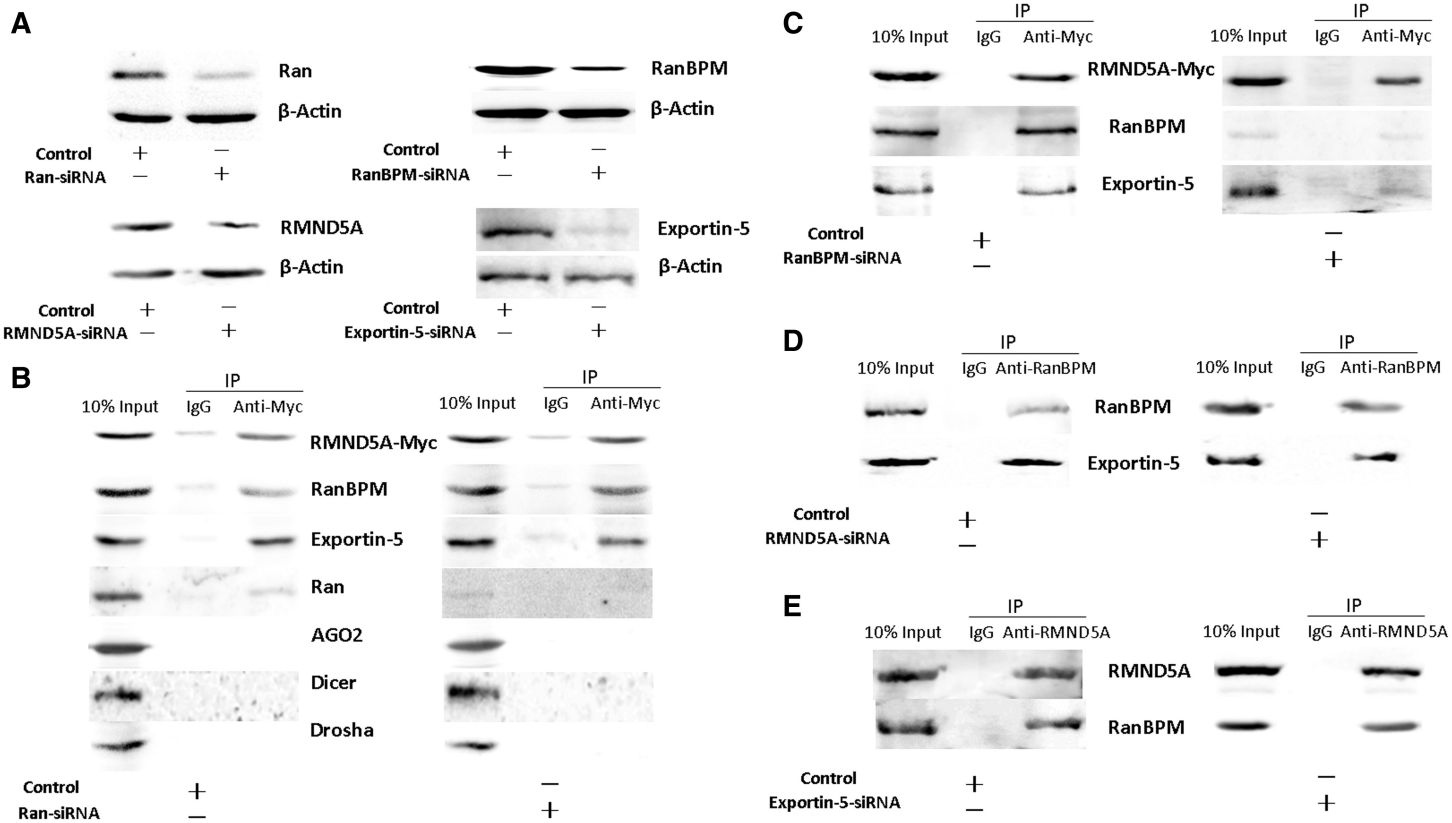
RMND5A interacts with Exportin-5. (**A**) HeLa cells were transfected with siRNA, and protein expression levels were determined after 48 h. Western blot analyses show that each specific siRNA downregulates the protein expression of Ran, RanBPM, RMND5A and Exportin-5 in HeLa cells. (**B**) RMND5A and Exportin-5 co-immunoprecipitation experiments. Full-length Myc-tagged RMND5A was overexpressed in HeLa cells. Anti-Myc antibody or normal rabbit IgG was added to 500 μg of HeLa cell lysate to immunoprecipitate protein complexes. Co-immunoprecipitation experiments were performed in HeLa cells following transfection with control (left panel) or Ran siRNA (right panel). (**C**) RMND5A and Exportin-5 co-immunoprecipitation experiments. Full-length Myc-tagged RMND5A was overexpressed in HeLa cells, and anti-Myc or normal rabbit IgG was added to 500 μg of HeLa cell lysates to immunoprecipitate protein complexes. Co-immunoprecipitation experiments were performed in HeLa cells transfected with control (left panel) or RanBPM siRNA (right panel). (**D**) RanBPM and Exportin-5 co-immunoprecipitation experiments. Anti-RanBPM or normal rabbit IgG was added to 500 μg HeLa cell lysates to immunoprecipitate protein complexes. Co-immunoprecipitation experiments were performed in HeLa cells transfected with control (left panel) or RMND5A siRNA (right panel). (**E**) RanBPM and RMND5A co-immunoprecipitation experiments. Anti-RMND5A or normal rabbit IgG was added to 500 μg of HeLa cell lysate samples to immunoprecipitate protein complexes. Co-immunoprecipitation experiments were performed in HeLa cells transfected with control (left panel) or Exportin-5 siRNA (right panel).

The human *RMND5A* gene, also known as *p44CTLH* or *RMD5*, is located on chromosome 2p11.2 and encodes a 44 kDa protein containing 391 amino acids. The RMND5A protein is composed of four domains: an N-terminal Lissencephaly type-1-like homology (LisH) motif, a C-terminal to LisH (CTLH) motif, a C-terminal CT11-RanBPM (CRA) motif and a Really Interesting New Gene (RING)-type zinc-finger motif (53). To identify the interaction between RMND5A and other proteins, a Myc-tagged RMND5A expression vector (pCDNA-RMND5A-Myc) was constructed using a commercial RMND5A cDNA expression plasmid (pCMV-RMND5A). We overexpressed RMND5A with Myc-tag and isolated the protein complex by co-immunoprecipitation. The eluted complex was analyzed by mass spectrometry (Supplementary Table S1 and Supplementary Figure S1). Two possible candidates bound by RMND5A had the highest score: Ran-binding protein in the microtubule-organizing center (RanBPM) and Exportin-5. Kobayashi *et al.* (54) previously reported that RMND5A directly interacts with RanBPM. As a scaffolding protein, RanBPM has been reported to weakly interact with Ran (55,56), which is essential for RNA and protein translocation through the nuclear pore complex. Exportin-5, encoded by the *XPO5* gene, depends on Ran-GTP to mediate the nucleocytoplasmic shuttling of miRNA precursors. The results of our co-immunoprecipitation experiments showed that Exportin-5, RanBPM and Ran were all present within the immune complex (Figure 2B). It appeared that Ran could be the hub for the interactions between RMND5A, RanBPM and Exportin-5, but our results did not corroborate this hypothesis. Our results indicate that the downregulation of Ran protein by a specific Ran siRNA (Figure 2A) does not affect RMND5A co-immunoprecipitation with Exportin-5 (Figure 2B). However, following RanBPM protein downregulation (Figure 2A), much less Exportin-5 was observed within the immune complex (Figure 2C). In addition, the interaction between RanBPM and Exportin-5 was not affected by the downregulation of RMND5A (Figure 2D), and the interaction between RMND5A and RanBPM was also not affected by Exportin-5 downregulation (Figure 2E). These results suggest that the interaction of RMND5A with Exportin-5 depends on RanBPM, but not on Ran, and RanBPM along is sufficient to interact with Exportin-5 or RMND5A. It was also interesting to find that other key proteins in miRNA biogenesis such as Drosha, Dicer and AGO2 do not appear in the complex of RanBPM and Exportin-5 when used the antibodies mentioned earlier in he text (Figure 2B).

**Figure 3.**
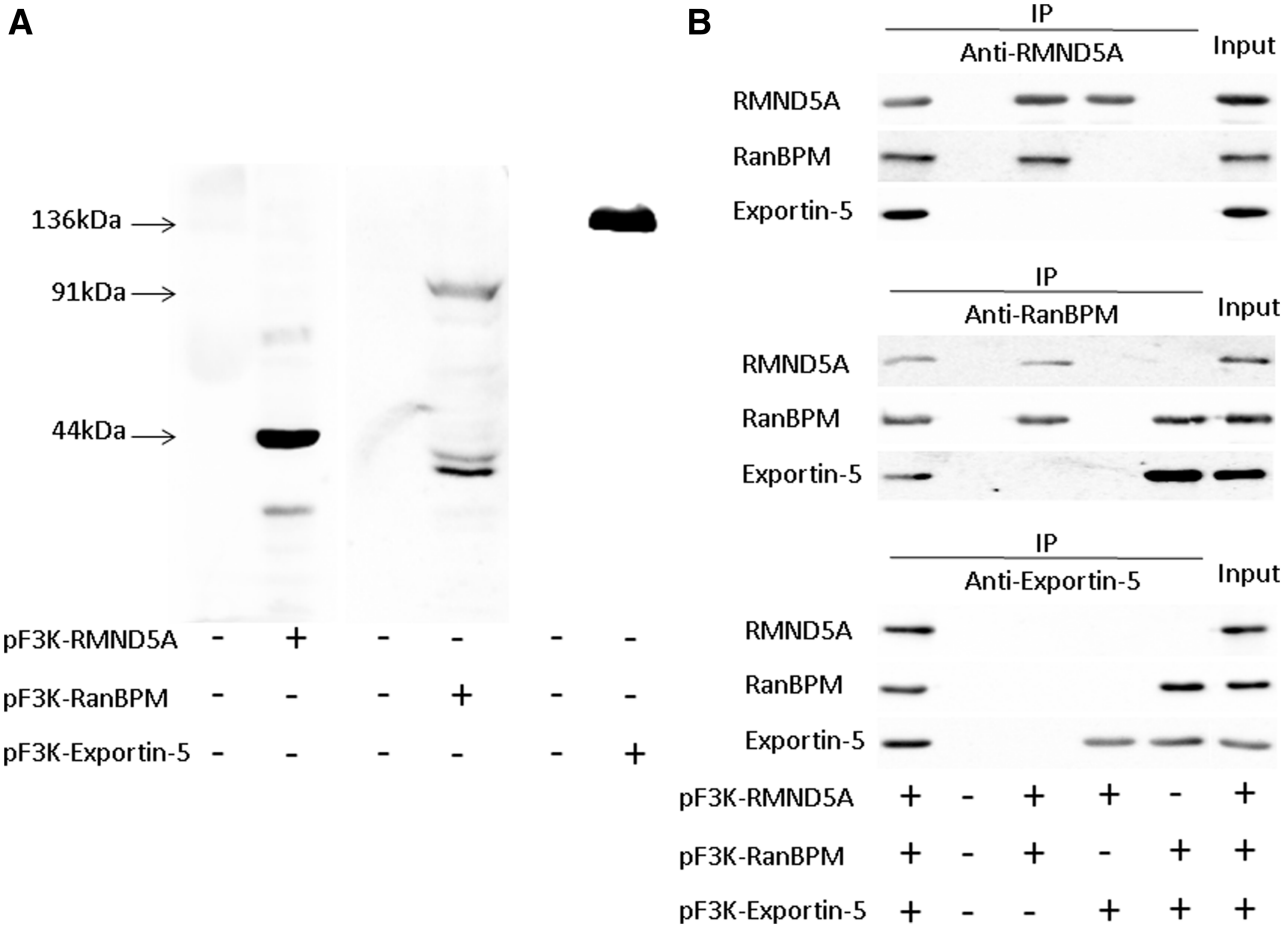
RMND5A interaction with RanBPM and Exportin-5 using an *in vitro* translation system. (**A**) RMND5A, RanBPM and Exportin-5 underwent *in vitro* translation separately with a TnT® sp6 High-Yield Wheat Germ Protein Express System. Each reaction mixture contained 3 μg of plasmid DNA that were incubated at 25°C for 2.5 h. Western blot analyses were performed to examine the expression proteins. RMND5A, 44 kDa; RanBPM, 91 kDa; Exportin-5, 136 kDa. (**B**) Co-immunoprecipitation experiments were performed using an *in vitro* translation system with anti-RMND5A, anti-RanBPM and anti-Exportin-5 antibody.

Next, RMND5A, RanBPM and Exportin-5 were expressed with the Wheat Germ Protein Expression System *in vitro* (Figure 3A). We further confirmed a direct interaction between RanBPM and RMND5A or Exportin-5. RMND5A could not interact with Exportin-5 in the absence of RanBPM (Figure 3B). In other words, RMND5A could not interact with Exportin-5 directly, and RanBPM appears to be the bridge for the interaction between RMND5A and Exportin-5.

**Figure 4.**
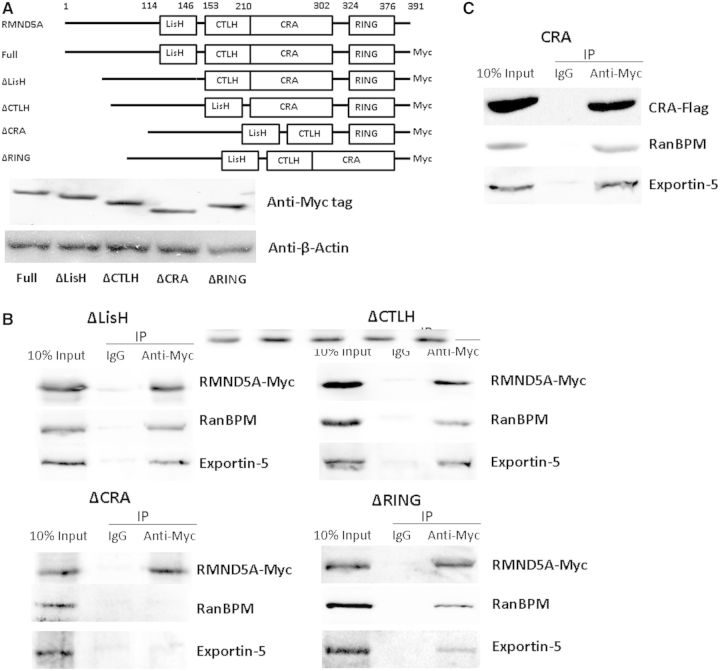
RMND5A interaction with RanBPM and Exportin-5 depends on a key structural domain. (**A**) RMND5A deletion mutant plasmids lacking each of the four structural domains (ΔLisH, ΔCTLH, ΔCRA and ΔRING) were constructed. Western blotting shows the expression of RMND5A deletion mutants. (**B**) Exportin-5 co-immunoprecipitates with RMND5A deletion mutants. Total cell protein was extracted from HeLa cells transfected with RMND5A ΔLisH expression plasmid. Co-immunoprecipitation experiments were performed with anti-Myc antibody or a normal rabbit IgG control. (**C**) Exportin-5 co-immunoprecipitates with the RMND5A CRA domain constructed with a Flag-tag and overexpressed. Total cell protein was extracted from HeLa cells transfected with the RMND5A ΔCTLH expression plasmid. Co-immunoprecipitation experiments were performed with anti-Myc antibody or a normal rabbit IgG control.

Next, we deleted four structural domains (LisH, CTLH, CRA and RING) of RMND5A and constructed Myc-tagged expression plasmids for the corresponding deletion mutants (Figure 4A). Co-immunoprecipitation results showed that RMND5A deletion mutants lacking LisH, CTLH or the RING structure domain could still interact with RanBPM and Exportin-5 (Figure 4B). However, the RMND5A deletion mutant lacking the CRA domain did not form a complex with RanBPM and Exportin-5 (Figure 4B). Further experiments showed that the CRA domain on its own was sufficient to precipitate RanBPM and Exportin-5 (Figure 4C). Thus, the RMND5A CRA domain appears to be necessary for RMND5A to interact with RanBPM and Exportin-5.

### MiR-138 targeting of RMND5A reduces Exportin-5 stability

**Figure 5.**
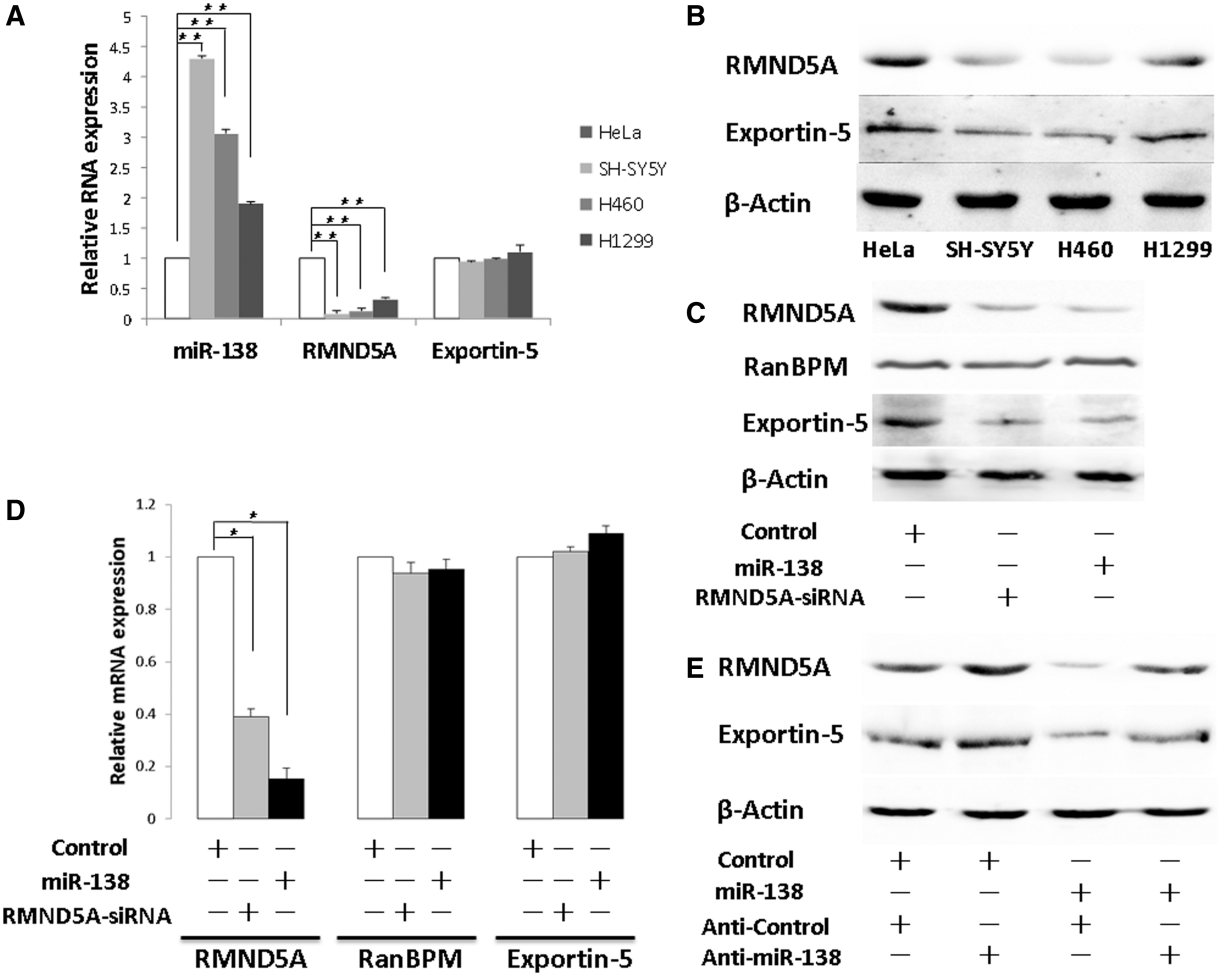
MiR-138 targeting of RMND5A reduces Exportin-5 protein levels. (**A**) Quantitative RT-PCR assays were performed to examine miR-138 and *RMND5A* mRNA levels in four cell types (HeLa, SH-SY5Y, H460 and H1299). GAPDH served as an internal control for the normalization of mRNA and U6 for that of miRNA. (**B**) Western blotting was performed to examine RMND5A and Exportin-5 protein levels in all four cell types. (**C**) Western blot analyses were performed to examine RMND5A, Exportin-5 and RanBPM protein levels in HeLa cells after treatment with miR-138 mimic or RMND5A siRNA for 48 h. (**D**) Quantitative RT-PCR assays were performed to examine *RMND5A XPO5* and *RanBPM* mRNA levels in HeLa cells treated with miR-138 mimic or RMND5A siRNA for 48 h. GAPDH served as an internal control. (**E**) Western blot analyses showed that miR-138 inhibitor (anti-miR-138) blocks the function of miR-138 and partially rescues Exportin-5 protein expression. Results are expressed as means ± s.d. for three independent experiments. *P < 0.05; **P < 0.01 versus control.

**Figure 6.**
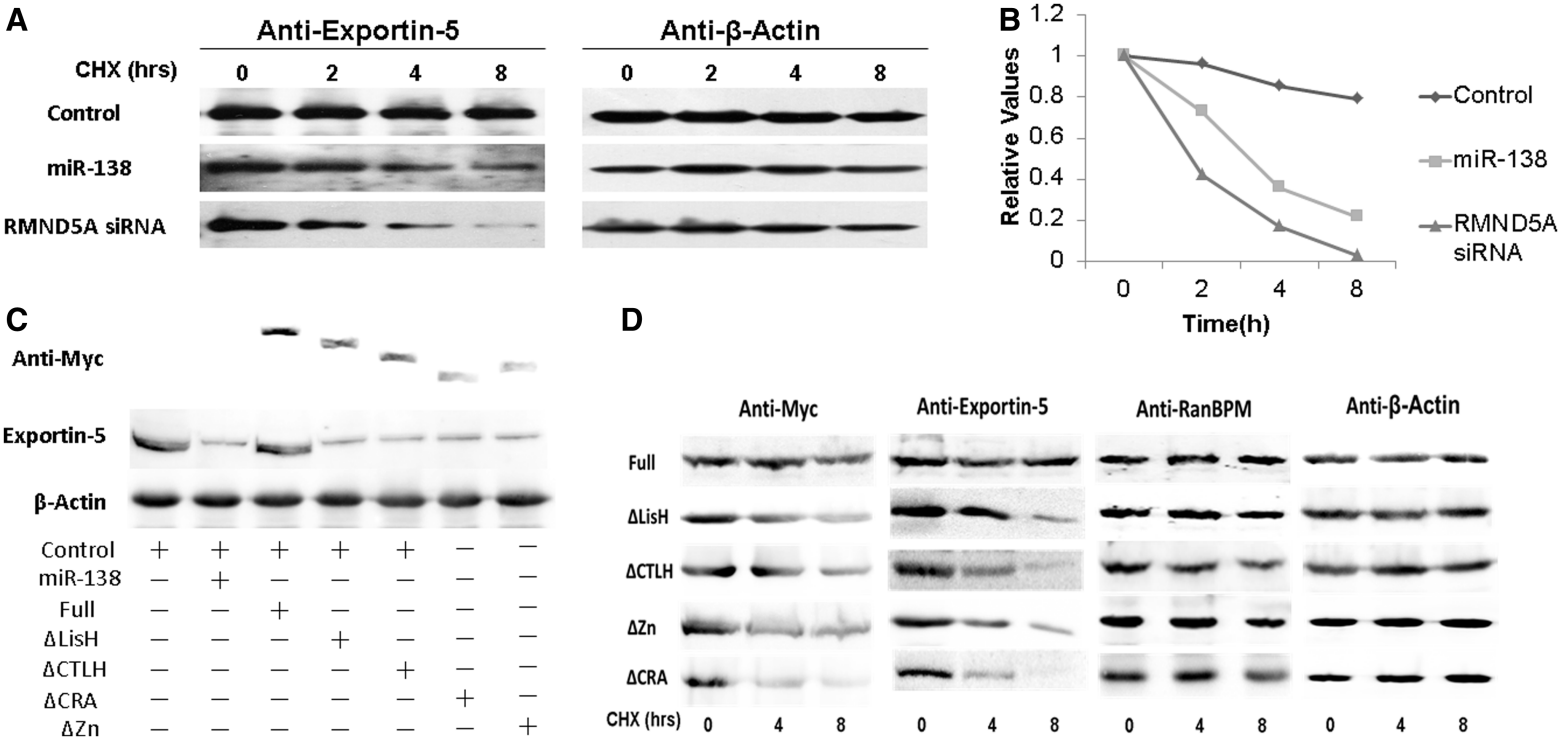
RMND5A stabilizes Exportin-5 protein in HeLa cells. (**A**) HeLa cells were transfected with Exportin-5 expression vectors, treated with cycloheximide and harvested at the indicated time points. The half-life of Exportin-5 protein was measured by western blotting. (**B**) Protein bands intensities were determined by densitometry using ImageJ software (rsb.info.nih.gov/ij), and Exportin-5 levels were normalized against β-actin. (**C**) HeLa cells were co-transfected with miR-138 mimic and full-length RMND5A or RMND5A deletion mutants for 48 h, treated with cycloheximide and then harvested after 8 h. Endogenous Exportin-5 protein expression was measured by western blotting. (**D**) HeLa cells were transfected with full-length RMND5A or RMND5A deletion mutants for 48 h. Cycloheximide (100 µg/ml) was added to the cells, and the cells were harvested after 0, 4 or 8 h. The stability of RMND5A wild-type and RMND5A deletion mutants were measured by western blotting.

In a comparative analysis, levels of miR-138 and *RMND5A* mRNA expression showed a negative correlation in four different cell types (HeLa, SH-SY5Y, H460 and H1299), which indicated that high miR-138 expression corresponded to low levels of *RMND5A*, and vice versa. *XPO5* mRNA was relatively stable in all four cell types, and there was no obvious difference in its expression among them (Figure 5A). However, it was surprising to discover that Exportin-5 protein expression also negatively correlated with miR-138 (Figure 5B). Next, using RMND5A siRNA as a positive control, we found that transfection with miR-138 mimic or RMND5A siRNA reduces endogenous RMND5A mRNA and protein levels. It was surprising to observe that Exportin-5 protein levels were also significantly downregulated (Figure 5C), although Exportin-5 mRNA levels were unaffected (Figure 5D). However, another factor in the complex, RanBPM, was not downregulated by miR-138 (Figure 5C and D). Cell treatments with a miR-138 inhibitor (anti-miR-138) effectively rescued RMND5A protein expression and partly recovered Exportin-5 expression (Figure 5E). Therefore, RMND5A knockdown has no effect on transcription but does affect protein levels of Exportin-5. These results prompted us to investigate whether the interaction with RMND5A inhibits Exportin-5 protein degradation. Using cycloheximide to inhibit protein synthesis, we found a clear increase in the rate of Exportin-5 degradation following transfection with miR-138 mimic and RMND5A siRNA (Figure 6A and B). In addition, normal levels of wild-type RMND5A were necessary to maintain endogenous Exportin-5 protein stability following miR-138 treatment. This ability was lost in all RMND5A deletion mutants (Figure 6C), although these rescue mutants achieved overexpression levels (Figure 4A). Furthermore, RMND5A deletion mutant proteins were less stable than the full-length protein, which might explain the apparent inability of deletion mutants to stabilize Exportin-5 (Figure 6D). In particular, the RMND5A deletion mutant that lacked the CRA domain was the most unstable, which suggested the CRA domain of RMND5A was important for its protein stability.

**Figure 7.**
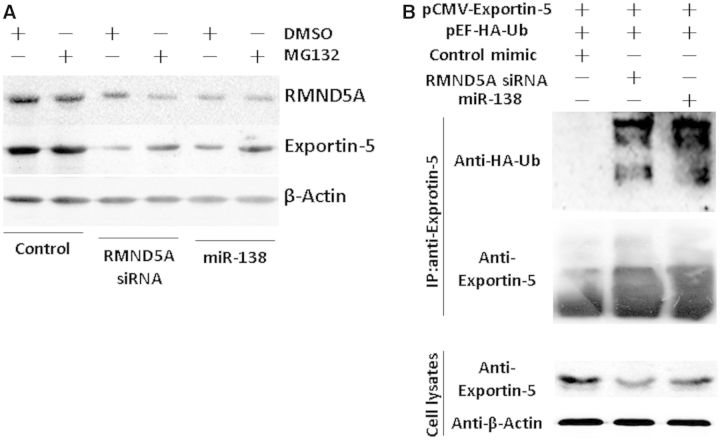
Detection of Exportin-5 ubiquitination in HeLa cells. (**A**) HeLa cells were transfected with control mimic, RMND5A siRNA or miR-138 mimic. After transfection for 36 h, cells were treated with proteasome inhibitor MG132 (20 μM) or Dimethyl sulfoxide (DMSO) for 8 h. Endogenous Exportin-5 protein expression was measured by western blotting. (**B**) HeLa cells were cotransfected with pCMV-Exportin-5, pEF-HA-Ub, control mimic (lane 1), RMND5A siRNA (lane 2) or miR-138 mimic (lane 3). Top: anti-Exportin-5 Immunoprecipitation pellets immunoblotted with anti-HA for Ub-Exportin-5 conjugates (upper), or anti-Exportin-5 to normalize the loading amounts (lower). Bottom: Immunoblotting of Exportin-5 protein levels in respective cell lysates, with β-actin serving as a loading control.

As RMND5A downregulation resulted in a rapid decrease of protein Exportin-5, this effect was partially abolished by the proteasome inhibitor MG132 (Figure 7A), indicating that RMND5A protect Exportin-5 protein against proteasome degradation. We also examined whether RMND5A affects ubiquitination of Exprotin-5 in HeLa cells. Results showed that the ubiquitination of Exportin-5 was confirmed and enhanced markedly by RMND5A knockdown by miR-138 or RMND5A siRNA in HeLa cells (Figure 7B). Taken together, these results demonstrated that RMND5A could protect against the ubiquitination and proteasome degradation of Exportin-5 protein.

**Figure 8.**
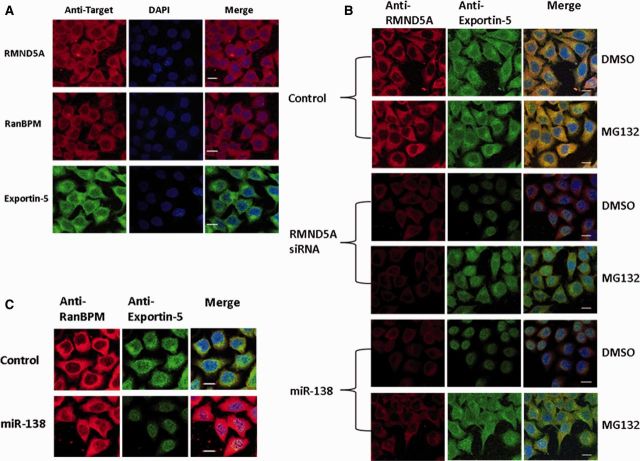
Characterization of RMND5A, RanBPM and Exporting-5 subcellular localization in HeLa cells. (**A**) RMND5A, RanBPM and Exporting-5 were detected by immunocytochemistry using specific antibodies. (**B**) HeLa cells were transfected with negative control mimic, miR-138 mimic or RMND5A siRNA. After transfection for 36 h, cells were treated with MG132 (20 μM) or DMSO for 8 h. RMND5A and Exportin-5 protein expression were analyzed by immunocytochemistry. Nuclei were counterstained with DAPI. (**C**) HeLa cells were transfected with negative control mimic or miR-138 mimic. RanBPM and Exportin-5 subcellular localization were detected by immunocytochemistry. Scale bar, 20 μm.

As RMND5A, RanBPM and Exportin-5 all shuttle between the nucleus and the cytoplasm (55,57), the identification of the complex and its subcellular localization will also be important. Confocal imaging analysis showed that Exportin-5 had a subcellular localization similar to that of RMND5A and RanBPM in HeLa cells, which all distributed throughout the nucleus and the cytoplasm but mainly in plasma surrounding the nucleus (Figure 8A). It was surprised to observe that Exportin-5 retention in nucleus when its protein levels were downregulated by RMND5A siRNA or miR-138. The proteasome inhibitor MG132 could recover not only Exportin-5 protein levels but also its normal subcellular localization (Figure 8B). However, RanBPM was not affected by miR-138 (Figure 8C).

Bennasser *et al.* (58) reported that the inhibition of Exportin-5 downregulates expression of Dicer by increasing the nuclear localization of *DICER* mRNA. In HeLa cells, the miR-138-mediated downregulation of Exportin-5 led to reduced levels of Dicer protein, and overexpression Exportin-5 effectively rescued Dicer protein expression (Supplementary Figure S2A). Four segments of the *DICER* 3′UTR and full-length *XPO5* 3′UTR were cloned into the luciferase reporter plasmid. Dual luciferase reporter assays showed that miR-138 transfection had no effect on *DICER* 3′UTR fragments or full-length *XPO5* 3′UTR (Supplementary Figure S2B). In addition, miR-138 expression did not affect *DICER* and *XPO5* mRNA expression (Supplementary Figure S2C). These results reveal that miR-138 influences Exportin-5 and Dicer protein levels by targeting RMND5A, and not by the direct targeting of *XPO5* and *DICER* mRNAs.

### MiR-138 inhibits the nuclear export of precursor miRNAs by downregulating RMND5A

**Figure 9.**
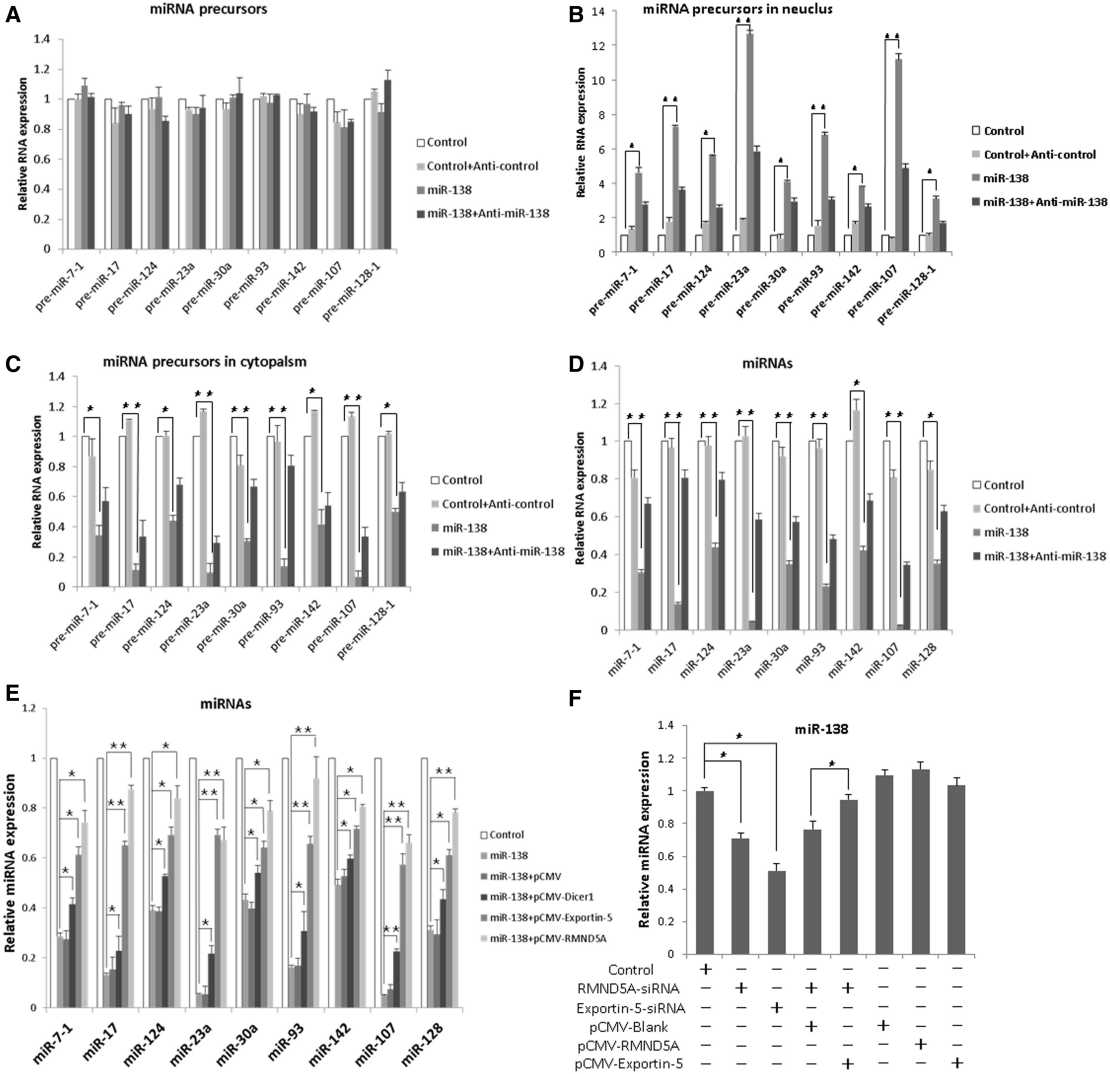
Mir-138 inhibits miRNA precursor processing in HeLa cells. (**A**) Quantitative RT-PCR assays were performed to examine the effects of miR-138 mimic and anti-miR-138 transfection on total cellular miRNA precursors. U6 served as an internal control. (**B**) Quantitative RT-PCR assays were performed to examine the effects of miR-138 mimic and anti-miR-138 transfection on cytoplasmic miRNA precursors. GAPDH mRNA was used for normalization. (**C**) Quantitative RT-PCR assays were performed to examine the effects of miR-138 mimic and anti-miR-138 transfection on nuclear miRNA precursors. U6 snRNA was used for normalization. (**D**) Mature miRNA expression was measured 48 h following transfection with miR-138 mimic. U6 snRNA was used as a control for normalization. (**E**) Mature miRNA expression was measured 48 h following co-transfection with miR-138 mimic and pCMV-Dicer, pCMV-Exportin-5 or pCMV-RMND5A. (**F**) Effect of knockdown or overexpression of RMND5A on miR-138 expression levels. HeLa cells were contransfected with control mimic, RMND5A siRNA, miR-138 mimic, pCMV-Blank or pCMV-Exportin-5. After transfection for 24 h, quantitative RT-PCR assays were performed to examine the expression levels of miR-138. U6 served as an internal control. *P < 0.05 and *P < 0.01. Data are representative of three independent experiments (mean ± s.d.).

Exportin-5 plays a key role in the nuclear transport of miRNA precursors, and Dicer is the RNase Ⅲ required for pre-miRNA maturation. Based on previous results, we speculated that RMND5A regulates miRNA biogenesis. To verify the miR-138-mediated inhibition of general miRNA processing through RMND5A targeting, the expression of individual miRNAs was measured in HeLa cells transfected with miR-138 mimic using a miRNA microarray. Microarray results revealed that the levels of 133 miRNAs were significantly altered by miR-138. These included 75 upregulated and 58 downregulated miRNAs (Supplementary Table S2). We selected nine miRNAs, including four with a relatively high abundance (miR-17, −30a, −107, −23a) and five with a low abundance (miR-142, −128, −7, −124, −93), which were all downregulated according to the microarray analysis. We therefore extracted total, nuclear and cytoplasmic RNA pools and measured the level of precursor miRNAs in each pool by qRT-PCR. The total amount of most of the selected miRNA precursors was not significant altered by treatment with miR-138 mimic or RMND5A siRNA (Figure 9A). However, our analysis showed that the expression of miRNA precursors were decreased in the cytoplasm and increased in the nucleus, thus indicating that precursor miRNAs are retained in the nucleus under these conditions (Figure 9B and C). Levels of the corresponding mature miRNAs were decreased in cells transfected with miR-138 mimic or RMND5A siRNA (Figure 9D). In HeLa cells treated with miR-138, overexpressed RMND5A, Dicer or Exportin-5 could partially restore miRNA expression levels (Figure 9E). It was interesting to find a reduction in miR-138 expression by knockdown RMND5A in HeLa cells. Overexpression of Exportin-5 restored miR-138 expression levels under RMND5A siRNA treatment (Figure 9F),which indicated a feedback loop in the pathway of miR-138, RMND5A and Exportin-5.

### MiR-138 inhibits HeLa cell migration

**Figure 10.**
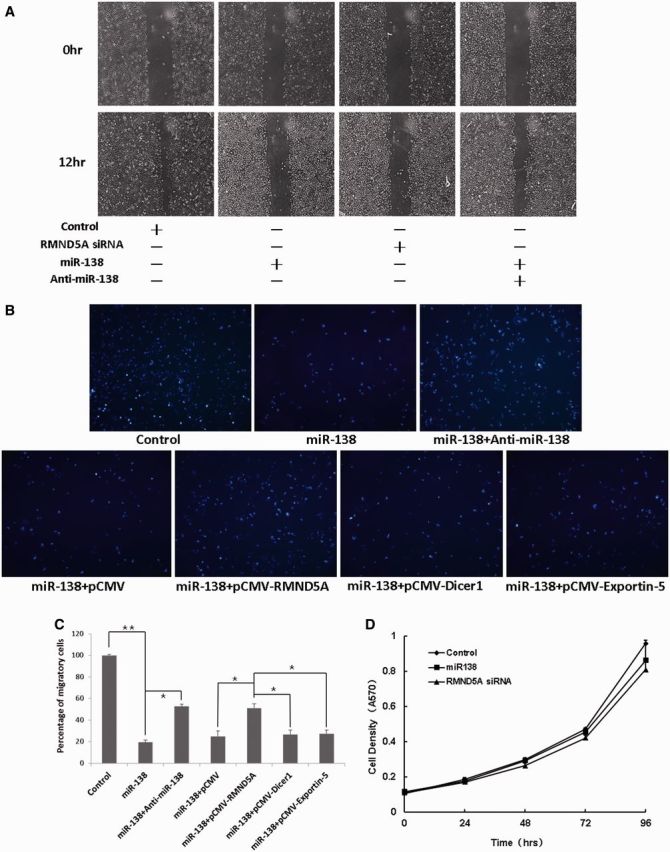
MiR-138 inhibits cell migration by downregulating RMND5A. (**A**) Migration of HeLa cells following transfection with miR-138 mimic or RMND5A siRNA using a wound-healing assay. After 12 h, migration of cells transfected with miR-138 mimic or RMND5A siRNA was significantly slower than in control cells. (**B**) Microscopic analysis of migrated HeLa cells stained with DAPI. HeLa cells were transiently transfected with a miR-138 mimic alone or co-transfected with pCMV-RMND5A, pCMV-Exportin-5 or pCMV-Dicer, and the motility of the transfected cells was evaluated using Transwell migration assays. (**C**) Relative cell migration was determined by the number of the DAPI-stained cells that migrated to the underside of the ﬁlter and normalized to the number of cells transfected with a control mimic. Cell migration is expressed as a percentage of that observed in the control and data are the means ± s.d. of three independent experiments. *P < 0.05; **P < 0.01 versus control. (**D**) MTT cell viability assay showed no difference in HeLa cell proliferation up to 96 h following transfection with miR-138 mimic or RMND5A siRNA. Each point represents the means ± s.d. Data are representative of three independent experiments.

RMND5A localizes to both the nucleus and the cytoplasm and is expressed in most human tissues, especially in the heart, liver and kidneys (59–61). The function of RMND5A was previously thought to relate to microtubule dynamics, cell migration, nuclear movement and chromosome segregation, for which the LisH and CTLH domains may play important roles. We next investigated the effect of miR-138 expression on HeLa cell migration using a wound healing assay. ‘Wounds’ were wider in cells treated with miR-138 mimic or RMND5A siRNA for 12 h than those treated with control siRNA (Figure 10A), which indicated that miR-138 inhibits the migration of HeLa cells. We obtained similar results using a Transwell migration assay, in which the number of cells transfected with miR-138 mimic or RMND5A siRNA that migrated to the basal side of the membrane was significantly lower than in controls (Figure 10B and C). HeLa cell migration could be effectively rescued by treatment with anti-miR-138 or RMND5A overexpression. However, the overexpression of Exportin-5 and Dicer could not restore HeLa cell migration. Next, we used siRNA to knock down Dicer and Exportin-5 protein expression and found that there was no clear relationship between Dicer or Exportin-5 and HeLa cell migration (Supplementary Figure S3). These data further suggest that the biological function of RMND5A was to regulate HeLa cell migration. Furthermore, using the MTT assay, we found that RMND5A knockdown did not significantly change HeLa cell proliferation, which suggested that there was no correlation between cell migration and proliferation (Figure 8D). Thus, miR-138 may inhibit cell migration through the inhibition of RMND5A.

## DISCUSSION

miRNAs can act as either tumor suppressors or promoters and therefore affect tumor development, proliferation, differentiation and many other pathological processes. The expression of miR-138 is generally low in tumors such as thyroid cancer, lung cancer, leukemia, HNSCC and TSCC. However, a correlation between miR-138 expression and cervical cancer has not been reported. Although the regulatory mechanism for maintaining low levels of miR-138 in tumors is undefined, there is a clear correlation between miR-138 expression and the development of many types of cancer. We first identified *RMND5A* as a miR-138 target in HeLa cells using bioinformatics and biological experiments. The previously reported miR-138 target genes, *RHOC* and *ROCK2*, inhibit TSCC cell migration and invasion (37). However, we found that miR-138 does not target *RHOC* and *ROCK2* in HeLa cells (Figure 1), but instead targets *RMND5A*. This indicates cell-type specificity in miRNA targeting. Little is currently known about the function of RMND5A. RMND5A contains a LisH/CTLH domain. Kobayashi *et al.* reported that RMND5A, RanBPM, Muskelin, p48EMLP, ARMC8α and ARMC8β are components of the CTLH complex. However, co-immunoprecipitation complexes analyzed by random mass spectrometry did not identify the other components of the CTLH complex, with the exception of RanBPM (Supplementary Table S1). Surprisingly, it was interesting to find nuclear transport receptors, including Exportin-5, Importin-4, Importin-7 and Exportin-1, in the co-immunoprecipitation complex, which mediate the nuclear export of RNA or protein in a RanGTP-dependent manner. However, our experiments showed that it was not Ran but RanBPM that mediated the non-direct interactions between Exportin-5 and RMND5A (Figures 4B, C and 5B). Further experiments revealed that miR-138 reduces the stability of Exportin-5 by targeting RMND5A, and thus decreasing RMND5A-regulated general miRNA expression. Thus, miR-138 is a negative regulator of miRNAs in HeLa cells. In turn, does RMND5A affect maturation of miR-138 itself or not? Indeed, almost a 30% reduction was observed in miR-138 expression levels by knockdown RMND5A in HeLa cells. Overexpressed Exportin-5 or RMND5A could not increase miR-138 expression levels versus control plasmid but could restore miR-138 expression levels under RMND5A siRNA treatment (Figure 8F). It strongly indicated a feedback loop in the pathway of miR-138, RMND5A and Exportin-5. However, it needs further work and good model to explore the dynamic change and balance among miR-138, RMND5A and Exportin-5.

As miR-138 reduces the stability of Exportin-5 and inhibits pre-miRNA nuclear export within HeLa cells, we first believed that the complex of RMNDA and Exportin-5 was formed and functioned in the nucleus. The results were interesting. Confocal imaging analysis showed that Exportin-5 had a subcellular localization similar to that of RMND5A and RanBPM in HeLa cells (Figure 9A), which indicated the complex can exist in cytoplasm or in nucleus, even shuttle from each other. Exportin-5 stranded in nucleus when its protein levels were indirectly downregulated by miR-138. As proteasome inhibitor MG132 treatment could recovery Exportin-5 normal subcellular localization (Figure 10B), it seemed that Exportin-5 active avoidance proteasome degradation from cytoplasm, and there might be other protectors in nucleus, such as RanGTP or pre-miRNAs.

MiR-138 directly targets RMND5A, but not Exportin-5 or Dicer, to influence HeLa cell migration (Figure 8A–C). There is controversy about the role of Dicer in cell migration. Martello *et al.* (62) reported that MDA-MB-231 cell migration was promoted following the miR-107-mediated inhibition of *DICER*; Tang *et al.* (63) reported that the knockdown of Dicer impairs the migratory capacity of HEK293T cells. Exportin-5 and Dicer are the key proteins in miRNA biogenesis, and miRNAs controlled by these proteins are likely to target genes that promote or inhibit cell migration. Although our experimental results showed that the individual levels of Dicer and Exportin-5 expression do not significantly correlate with HeLa cell migration, it may be that their relative balance affects cell migration in the specific environment of HeLa cells. Therefore, we hypothesize that miR-138 mimic inhibit HeLa cell migration, possibly via a complex multistep process that includes the inhibition of RMND5A protein function and changes in general miRNA expression. These processes may be particularly associated with the development of cervical carcinoma.

**Figure 11.**
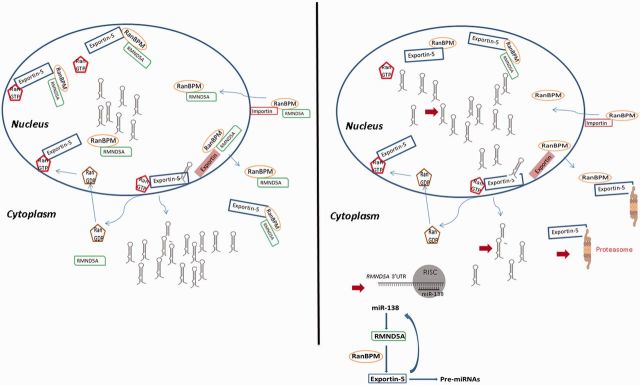
Model outlining the role of miR-138 and RMND5A in miRNA processing pathway in HeLa cells.

Further work will be required to explore the relationship between RMND5A and other nuclear transport receptors, including Importin-4, Importin-7 and Exportin-1, which were evident in the current mass spectrometry analysis results. It also needs to be confirmed whether RanBPM is the mediatory protein for these interactions and the function of these complexes. In summary, we have uncovered a new function for miR-138 and RMND5A and identified an miRNA processing pathway that is regulated by miR-138 in HeLa cells (Figure 11). We find miR-138 target RMND5A is a common phenomenon in other cancer cells such as human airway cell line and TSCC cell line by gathering information from publicly available microarray data in GEO (Supplementary Figure S4). It will be interesting to analyze whether miR-138-RMND5A-Exportin-5 pathway function in other biological processes associated with miR-138 such as miR-138 modulating DNA damage response by repressing histone H2AX (45). It will therefore be interesting to identify other miRNAs that directly or indirectly affect key proteins involved in miRNA biogenesis that regulate miRNA processing (62) in a similar manner to the miR-138-mediated inhibition of Exportin-5 stability.

## SUPPLEMENTARY DATA

Supplementary Data are available at NAR Online.

## FUNDING

Funding for open access charge: Major State Basic Research Development Program of China (973 Program) [2011CB811300, 2010CB912801] and National Natural Science Foundation of China [Project Nos. 31100569, 30971630].

*Conflict of interest statement*. None declared.

## Supplementary Material

Supplementary Data

## References

[1] Bartel DP (2004). MicroRNAs: genomics, biogenesis, mechanism, and function. Cell.

[2] Djuranovic S, Nahvi A, Green R (2011). A parsimonious model for gene regulation by miRNAs. Science.

[3] Filipowicz W, Bhattacharyya SN, Sonenberg N (2008). Mechanisms of post-transcriptional regulation by microRNAs: are the answers in sight. Nat. Rev. Genet..

[4] Kusenda B, Mraz M, Mayer J, Pospisilova S (2006). MicroRNA biogenesis, functionality and cancer relevance. Biomed. Pap. Med. Fac. Univ. Palacky Olomouc Czech Repub..

[5] Lim LP, Lau NC, Garrett-Engele P, Grimson A, Schelter JM, Castle J, Bartel DP, Linsley PS, Johnson JM (2005). Microarray analysis shows that some microRNAs downregulate large numbers of target mRNAs. Nature.

[6] Brennecke J, Hipfner DR, Stark A, Russell RB, Cohen SM (2003). bantam encodes a developmentally regulated microRNA that controls cell proliferation and regulates the proapoptotic gene hid in Drosophila. Cell.

[7] Cuellar TL, McManus MT (2005). MicroRNAs and endocrine biology. J. Endocrinol..

[8] Poy MN, Eliasson L, Krutzfeldt J, Kuwajima S, Ma X, Macdonald PE, Pfeffer S, Tuschl T, Rajewsky N, Rorsman P (2004). A pancreatic islet-specific microRNA regulates insulin secretion. Nature.

[9] Chen CZ, Li L, Lodish HF, Bartel DP (2004). MicroRNAs modulate hematopoietic lineage differentiation. Science.

[10] Wilfred BR, Wang WX, Nelson PT (2007). Energizing miRNA research: a review of the role of miRNAs in lipid metabolism, with a prediction that miR-103/107 regulates human metabolic pathways. Mol. Genet. Metab..

[11] Ha TY (2011). MicroRNAs in human diseases: from lung, liver and kidney diseases to infectious disease, sickle cell disease and Endometrium disease. Immune Netw..

[12] Martignani E, Miretti S, Accornero P, Baratta M (2011). miRNAs highlights in stem and cancer cells. Mini Rev. Med. Chem..

[13] Iorio MV, Croce CM (2012). MicroRNA dysregulation in cancer: diagnostics, monitoring and therapeutics. EMBO Mol. Med..

[14] Ivey KN, Muth A, Arnold J, King FW, Yeh RF, Fish JE, Hsiao EC, Schwartz RJ, Conklin BR, Bernstein HS (2008). MicroRNA regulation of cell lineages in mouse and human embryonic stem cells. Cell Stem Cell.

[15] Sun G, Li H, Wu X, Covarrubias M, Scherer L, Meinking K, Luk B, Chomchan P, Alluin J, Gombart AF (2012). Interplay between HIV-1 infection and host microRNAs. Nucleic Acids Res..

[16] Zampetaki A, Mayr M (2012). MicroRNAs in vascular and metabolic disease. Circ. Res..

[17] Bartel DP (2009). MicroRNAs: target recognition and regulatory functions. Cell.

[18] Cai X, Hagedorn CH, Cullen BR (2004). Human microRNAs are processed from capped, polyadenylated transcripts that can also function as mRNAs. RNA.

[19] Lee Y, Kim M, Han J, Yeom KH, Lee S, Baek SH, Kim VN (2004). MicroRNA genes are transcribed by RNA polymerase II. EMBO J..

[20] Faller M, Guo F (2008). MicroRNA biogenesis: there's more than one way to skin a cat. Biochim. Biophys. Acta.

[21] Rodriguez A, Griffiths-Jones S, Ashurst JL, Bradley A (2004). Identification of mammalian microRNA host genes and transcription units. Genome Res..

[22] Lee Y, Ahn C, Han J, Choi H, Kim J, Yim J, Lee J, Provost P, Radmark O, Kim S (2003). The nuclear RNase Ⅲ Drosha initiates microRNA processing. Nature.

[23] Gregory RI, Chendrimada TP, Shiekhattar R (2006). MicroRNA biogenesis: isolation and characterization of the microprocessor complex. Methods Mol. Biol..

[24] Yi R, Qin Y, Macara IG, Cullen BR (2003). Exportin-5 mediates the nuclear export of pre-microRNAs and short hairpin RNAs. Genes Dev..

[25] Lund E, Guttinger S, Calado A, Dahlberg JE, Kutay U (2004). Nuclear export of microRNA precursors. Science.

[26] Bohnsack MT, Czaplinski K, Gorlich D (2004). Exportin 5 is a RanGTP-dependent dsRNA-binding protein that mediates nuclear export of pre-miRNAs. RNA.

[27] Yi R, Doehle BP, Qin Y, Macara IG, Cullen BR (2005). Overexpression of exportin 5 enhances RNA interference mediated by short hairpin RNAs and microRNAs. RNA.

[28] Lund E, Dahlberg JE (2006). Substrate selectivity of exportin 5 and Dicer in the biogenesis of microRNAs. Cold Spring Harb. Symp. Quant. Biol..

[29] Macrae IJ, Zhou K, Li F, Repic A, Brooks AN, Cande WZ, Adams PD, Doudna JA (2006). Structural basis for double-stranded RNA processing by Dicer. Science.

[30] Gregory RI, Chendrimada TP, Cooch N, Shiekhattar R (2005). Human RISC couples microRNA biogenesis and posttranscriptional gene silencing. Cell.

[31] Rana TM (2007). Illuminating the silence: understanding the structure and function of small RNAs. Nat. Rev. Mol. Cell Biol..

[32] Lagos-Quintana M, Rauhut R, Yalcin A, Meyer J, Lendeckel W, Tuschl T (2002). Identification of tissue-specific microRNAs from mouse. Curr. Biol..

[33] Griffiths-Jones S (2004). The microRNA Registry. Nucleic Acids Res..

[34] Weber MJ (2005). New human and mouse microRNA genes found by homology search. FEBS J..

[35] Liu X, Jiang L, Wang A, Yu J, Shi F, Zhou X (2009). MicroRNA-138 suppresses invasion and promotes apoptosis in head and neck squamous cell carcinoma cell lines. Cancer Lett..

[36] Liu X, Wang C, Chen Z, Jin Y, Wang Y, Kolokythas A, Dai Y, Zhou X (2011). MicroRNA-138 suppresses epithelial-mesenchymal transition in squamous cell carcinoma cell lines. Biochem. J..

[37] Jiang L, Liu X, Kolokythas A, Yu J, Wang A, Heidbreder CE, Shi F, Zhou X (2010). Downregulation of the Rho GTPase signaling pathway is involved in the microRNA-138-mediated inhibition of cell migration and invasion in tongue squamous cell carcinoma. Int. J. Cancer.

[38] Yeh YM, Chuang CM, Chao KC, Wang LH (2013). MicroRNA-138 suppresses ovarian cancer cell invasion and metastasis by targeting SOX4 and HIF-1alpha. Int. J. Cancer.

[39] Jiang L, Dai Y, Liu X, Wang C, Wang A, Chen Z, Heidbreder CE, Kolokythas A, Zhou X (2011). Identification and experimental validation of G protein alpha inhibiting activity polypeptide 2 (GNAI2) as a microRNA-138 target in tongue squamous cell carcinoma. Hum. Genet..

[40] Jin Y, Wang C, Liu X, Mu W, Chen Z, Yu D, Wang A, Dai Y, Zhou X (2011). Molecular characterization of the microRNA-138-Fos-like antigen 1 (FOSL1) regulatory module in squamous cell carcinoma. J. Biol. Chem..

[41] Siegel G, Obernosterer G, Fiore R, Oehmen M, Bicker S, Christensen M, Khudayberdiev S, Leuschner PF, Busch CJ, Kane C (2009). A functional screen implicates microRNA-138-dependent regulation of the depalmitoylation enzyme APT1 in dendritic spine morphogenesis. Nat. Cell Biol..

[42] Yang Z, Bian C, Zhou H, Huang S, Wang S, Liao L, Zhao RC (2011). MicroRNA hsa-miR-138 inhibits adipogenic differentiation of human adipose tissue-derived mesenchymal stem cells through adenovirus EID-1. Stem Cells Dev..

[43] Gong H, Song L, Lin C, Liu A, Lin X, Wu J, Li M, Li J (2013). Downregulation of miR-138 sustains NF-kappaB activation and promotes lipid raft formation in esophageal squamous cell carcinoma. Clin. Cancer Res..

[44] Eskildsen T, Taipaleenmaki H, Stenvang J, Abdallah BM, Ditzel N, Nossent AY, Bak M, Kauppinen S, Kassem M (2011). MicroRNA-138 regulates osteogenic differentiation of human stromal (mesenchymal) stem cells *in vivo*. Proc. Natl Acad. Sci. USA.

[45] Wang Y, Huang JW, Li M, Cavenee WK, Mitchell PS, Zhou X, Tewari M, Furnari FB, Taniguchi T (2011). MicroRNA-138 modulates DNA damage response by repressing histone H2AX expression. Mol. Cancer Res..

[46] Mitomo S, Maesawa C, Ogasawara S, Iwaya T, Shibazaki M, Yashima-Abo A, Kotani K, Oikawa H, Sakurai E, Izutsu N (2008). Downregulation of miR-138 is associated with overexpression of human telomerase reverse transcriptase protein in human anaplastic thyroid carcinoma cell lines. Cancer Sci..

[47] Zhao X, Yang L, Hu J, Ruan J (2010). miR-138 might reverse multidrug resistance of leukemia cells. Leuk. Res..

[48] Wong TS, Liu XB, Chung-Wai HA, Po-Wing YA, Wai-Man NR, Ignace WW (2008). Identification of pyruvate kinase type M2 as potential oncoprotein in squamous cell carcinoma of tongue through microRNA profiling. Int. J. Cancer.

[49] Wong TS, Liu XB, Wong BY, Ng RW, Yuen AP, Wei WI (2008). Mature miR-184 as Potential Oncogenic microRNA of Squamous Cell Carcinoma of Tongue. Clin. Cancer Res..

[50] Obernosterer G, Leuschner PJ, Alenius M, Martinez J (2006). Post-transcriptional regulation of microRNA expression. RNA.

[51] Huang BX, Kim HY (2013). Effective identification of Akt interacting proteins by two-step chemical crosslinking, co-immunoprecipitation and mass spectrometry. PLoS One.

[52] Choo YS, Zhang Z (2009). Detection of protein ubiquitination. J Vis Exp..

[53] Strausberg RL, Feingold EA, Grouse LH, Derge JG, Klausner RD, Collins FS, Wagner L, Shenmen CM, Schuler GD, Altschul SF (2002). Generation and initial analysis of more than 15,000 full-length human and mouse cDNA sequences. Proc. Natl Acad. Sci. USA.

[54] Kobayashi N, Yang J, Ueda A, Suzuki T, Tomaru K, Takeno M, Okuda K, Ishigatsubo Y (2007). RanBPM, Muskelin, p48EMLP, p44CTLH, and the armadillo-repeat proteins ARMC8alpha and ARMC8beta are components of the CTLH complex. Gene.

[55] Nishitani H, Hirose E, Uchimura Y, Nakamura M, Umeda M, Nishii K, Mori N, Nishimoto T (2001). Full-sized RanBPM cDNA encodes a protein possessing a long stretch of proline and glutamine within the N-terminal region, comprising a large protein complex. Gene.

[56] Suresh B, Ramakrishna S, Baek KH (2012). Diverse roles of the scaffolding protein RanBPM. Drug Discov. Today.

[57] Brownawell AM, Macara IG (2002). Exportin-5, a novel karyopherin, mediates nuclear export of double-stranded RNA binding proteins. J. Cell Biol..

[58] Bennasser Y, Chable-Bessia C, Triboulet R, Gibbings D, Gwizdek C, Dargemont C, Kremer EJ, Voinnet O, Benkirane M (2011). Competition for XPO5 binding between Dicer mRNA, pre-miRNA and viral RNA regulates human Dicer levels. Nat. Struct. Mol. Biol..

[59] Yan W, Lee H, Yi EC, Reiss D, Shannon P, Kwieciszewski BK, Coito C, Li XJ, Keller A, Eng J (2004). System-based proteomic analysis of the interferon response in human liver cells. Genome Biol..

[60] Resing KA, Meyer-Arendt K, Mendoza AM, Aveline-Wolf LD, Jonscher KR, Pierce KG, Old WM, Cheung HT, Russell S, Wattawa JL (2004). Improving reproducibility and sensitivity in identifying human proteins by shotgun proteomics. Anal. Chem..

[61] Tanner S, Shen Z, Ng J, Florea L, Guigo R, Briggs SP, Bafna V (2007). Improving gene annotation using peptide mass spectrometry. Genome Res..

[62] Martello G, Rosato A, Ferrari F, Manfrin A, Cordenonsi M, Dupont S, Enzo E, Guzzardo V, Rondina M, Spruce T (2010). A MicroRNA targeting dicer for metastasis control. Cell.

[63] Tang KF, Song GB, Shi YS, Yuan L, Li YH (2010). Dicer knockdown induces fibronectin-1 expression in HEK293T cells via induction of Egr1. Biochim. Biophys. Acta.

