# Supplementary material for: MiR-138 downregulates miRNA processing in HeLa cells by targeting RMND5A and decreasing Exportin-5 stability
Source: Nucleic Acids Res. 2013 Sep 19;42(1):458–74. doi: 10.1093/nar/gkt839 (PMC3874158; doi:10.1093/nar/gkt839)
Supplement: Supplementary Data [file supp_42_1_458__index.html]

MiR-138 downregulates miRNA processing in HeLa cells by targeting RMND5A and decreasing Exportin-5 stability — MiR-138 downregulates miRNA processing in HeLa cells by targeting RMND5A and decreasing Exportin-5 stability — Supplementary Data 

# MiR-138 downregulates miRNA processing in HeLa cells by targeting *RMND5A* and decreasing Exportin-5 stability

## Supplementary Data

files

**Files in this Data Supplement:**

- Supplementary Data - doc file
