# Supplementary material for: MiR-138 downregulates miRNA processing in HeLa cells by targeting RMND5A and decreasing Exportin-5 stability
Source: Nucleic Acids Res. 2013 Sep 19;42(1):458–74. doi: 10.1093/nar/gkt839 (PMC3874158; doi:10.1093/nar/gkt839)
Supplement: Supplementary Data [file supp_gkt839_nar-01408-y-2013-File013.doc]

**Supplementary Table1：Differentially regulated genes by miR-138^a^** in HeLa cells

| **log2 (Ratio of miR-138/control)** | **P-value (Differentially expressed)** | **Gene symbol** |
| --- | --- | --- |
| 1.316643 | 0.017519 | FBN1 |
| 1.776156 | 0.013405 | ZBTB5 |
| 1.010614 | 0.036333 | MGAT5 |
| 1.578691 | 0.008232 | FBXL5 |
| 1.294618 | 0.037695 | FUNDC1 |
| 1.277008 | 0.033489 | HIST1H1C |
| 1.165803 | 0.021414 | CCM2 |
| 1.231518 | 0.048625 | BECN1 |
| 1.723995 | 0.005801 | ZW10 |
| 1.004862 | 0.028851 | CCDC104 |
| 1.829825 | 0.000998 | PRMT6 |
| 1.264643 | 0.049103 | GSTZ1 |
| 1.12725 | 0.024795 | APBA2 |
| 1.231957 | 0.029266 | AK3 |
| 1.442019 | 0.00444 | EPDR1 |
| 1.179737 | 0.016319 | GOLT1B |
| 1.255257 | 0.037678 | YIPF4 |
| 1.438276 | 0.012774 | OGFR |
| 1.119277 | 0.031004 | GDI1 |
| 1.227425 | 0.011538 | NOLC1 |
| 1.377839 | 0.029185 | GART |
| 1.205854 | 0.010808 | PRDX4 |
| 1.081981 | 0.023189 | CSE1L |
| 1.128 | 0.031415 | CLCC1 |
| 1.233275 | 0.031711 | PAFAH1B2 |
| 1.064159 | 0.041892 | MINPP1 |
| 1.328989 | 0.007737 | HOXB6 |
| 1.086741 | 0.04109 | DHX37 |
| 1.162555 | 0.021941 | NDUFB6 |
| 1.180376 | 0.045954 | CD72 |
| 1.7934 | 0.020268 | POMT2 |
| 1.149438 | 0.032527 | CYB561D2 |
| 1.001896 | 0.031353 | STK25 |
| 1.138261 | 0.049387 | TBC1D9B |
| 1.246607 | 0.017032 | C4orf3 |
| 1.245669 | 0.011008 | PSMD14 |
| 2.466973 | 0.000076 | HIST1H2BD |
| 1.113004 | 0.035835 | BPIFB2 |
| 1.233301 | 0.022475 | NRAS |
| 1.144576 | 0.029082 | ACSL3 |
| 1.730325 | 0.001074 | TOMM22 |
| 1.055136 | 0.032038 | SLC33A1 |
| 1.195929 | 0.029955 | ERLIN1 |
| 1.404617 | 0.013222 | TM2D3 |
| 1.133491 | 0.022576 | PPM1K |
| 1.200247 | 0.017447 | R3HCC1 |
| 1.422618 | 0.00355 | HABP4 |
| 1.583041 | 0.036259 | LRP12 |
| 1.059039 | 0.024849 | LOC100128822 |
| 1.283713 | 0.007421 | PLEKHB2 |
| 1.127564 | 0.028816 | UTP3 |
| 1.046946 | 0.021554 | WDR54 |
| 1.074248 | 0.033734 | DLK2 |
| 1.325329 | 0.006822 | TM4SF1 |
| 1.152431 | 0.032142 | CALR |
| 1.292955 | 0.007516 | MT1X |
| 1.261296 | 0.022121 | POP7 |
| 1.177185 | 0.016564 | RGS19 |
| 1.673482 | 0.005303 | STX12 |
| 1.293947 | 0.009591 | YIPF6 |
| 1.399447 | 0.028034 | SPTLC1 |
| 1.41732 | 0.005342 | CETN3 |
| 1.009735 | 0.037939 | FAM114A1 |
| 1.275613 | 0.009536 | POMP |
| 1.869118 | 0.000745 | G3BP2 |
| 1.132395 | 0.028293 | INTS8 |
| 1.071279 | 0.02247 | ZNF227 |
| 1.208811 | 0.046127 | HDAC3 |
| 1.478649 | 0.004723 | CTGF |
| 1.710506 | 0.01317 | SLC2A3 |
| 1.044601 | 0.035154 | OXR1 |
| 1.411894 | 0.005581 | C15orf57 |
| 1.464238 | 0.004915 | FNBP1 |
| 1.12846 | 0.026325 | NOSIP |
| 1.306229 | 0.012073 | NFE2L1 |
| 1.249868 | 0.010954 | NDUFS2 |
| 1.344633 | 0.007206 | SLC39A7 |
| 1.071345 | 0.031214 | INSIG1 |
| 1.196845 | 0.019648 | POLR2D |
| 1.376524 | 0.03583 | AIMP1 |
| 1.656474 | 0.008977 | SDC4 |
| 1.238863 | 0.041871 | SUMF1 |
| 1.581364 | 0.049476 | ZNF91 |
| 1.002479 | 0.045662 | HSP90B3P|HSP90B1 |
| 1.682665 | 0.001761 | TDG|LOC732360 |
| 1.429487 | 0.017113 | MRFAP1L1 |
| 1.118059 | 0.022272 | MORF4L1 |
| 1.048229 | 0.047421 | PPT1 |
| 1.311639 | 0.011044 | GOLGA2 |
| 1.424938 | 0.021141 | MED31 |
| 1.483439 | 0.004237 | TMEM30A |
| 1.427045 | 0.004755 | SBDS|SBDSP1 |
| 1.090612 | 0.026791 | PERP |
| 1.019911 | 0.034382 | DCBLD2 |
| 1.110935 | 0.024258 | C4orf3 |
| 1.194514 | 0.015398 | TXNDC12 |
| 1.068612 | 0.049681 | WDR26 |
| 1.317656 | 0.006456 | MRFAP1 |
| 1.652184 | 0.038552 | RAC1 |
| 1.731358 | 0.002895 | TOMM22 |
| 1.459775 | 0.007025 | LOC147727 |
| 1.083927 | 0.024765 | COX17 |
| 1.015223 | 0.034836 | KLC1 |
| 1.04517 | 0.029394 | MESDC2 |
| 1.613189 | 0.005508 | MSRB3 |
| 1.42566 | 0.006643 | GTF2H5 |
| 1.083765 | 0.022878 | PREPL |
| 1.141964 | 0.030184 | TOP1 |
| 1.14786 | 0.047235 | IER3IP1 |
| 2.348478 | 0.012539 | TIPIN |
| 2.349911 | 0.000628 | PAFAH1B2 |
| 1.399403 | 0.018578 | SRPX2 |
| 1.069888 | 0.044323 | TTC32 |
| 1.164202 | 0.042185 | SYCE3 |
| 1.027953 | 0.027155 | MMADHC |
| 1.340959 | 0.012661 | GOLGA2 |
| 1.21174 | 0.013405 | NEFH |
| 2.105193 | 0.001686 | EIF3L |
| 1.39377 | 0.014949 | HDAC1 |
| 1.128971 | 0.019888 | USP39 |
| 1.004517 | 0.035094 | MATR3 |
| 1.240025 | 0.024545 | RRBP1 |
| 1.017073 | 0.031899 | SGCE |
| 1.159302 | 0.012982 | CD164 |
| 1.717077 | 0.004036 | EEF1E1 |
| 1.340419 | 0.026631 | CCBL2 |
| 1.780159 | 0.001523 | HIST1H2BD |
| 1.141964 | 0.029934 | SEC22C |
| 1.334984 | 0.042342 | TTLL9 |
| 1.027488 | 0.032485 | TADA3 |
| 1.40396 | 0.00968 | XPC |
| 1.859523 | 0.000657 | C7orf55 |
| 1.471085 | 0.047171 | ZNF721 |
| 1.31579 | 0.024284 | TRAK1 |
| 1.590516 | 0.007036 | NA |
| 1.196765 | 0.014987 | LOC100128979 |
| 1.091679 | 0.032642 | ISPD |
| 1.335841 | 0.032303 | NME1 |
| 1.242613 | 0.008288 | FDPS |
| 1.075119 | 0.019871 | HSPB1 |
| 1.006265 | 0.034054 | STYX |
| 1.498661 | 0.003011 | PRDX4 |
| 1.225051 | 0.015434 | MTCH1 |
| 1.287404 | 0.015794 | PIGY |
| 1.568976 | 0.016097 | ZNF702P |
| 1.111055 | 0.038164 | PROSC |
| 1.223275 | 0.036301 | ZNF252 |
| 1.017926 | 0.025593 | NOLC1 |
| 1.144377 | 0.013594 | INSIG1 |
| 1.846526 | 0.003155 | SETD7 |
| 1.293777 | 0.018641 | PSMC3 |
| 1.520806 | 0.007414 | HABP4 |
| 1.4599 | 0.01501 | AFF4 |
| 1.453027 | 0.009414 | CAB39L |
| 1.228408 | 0.040689 | PRKAB2 |
| 1.379587 | 0.029826 | TMX3 |
| 1.967611 | 0.00045 | BICD2 |
| 1.918533 | 0.015438 | PHKB |
| 1.249642 | 0.012754 | MAGEA4 |
| 1.285167 | 0.012757 | EIF4A2 |
|  |  | **Gene_symbol** |
| -1.007844 | 0.026168 | GOT1 |
| -1.127488 | 0.045689 | ITPKA |
| -1.361059 | 0.016873 | PHGDH |
| -1.822569 | 0.000755 | ST6GALNAC4 |
| -1.409293 | 0.004481 | SESN2 |
| -1.28945 | 0.006822 | ISOC1 |
| -1.160467 | 0.017657 | SH2B3 |
| -1.140901 | 0.048807 | ETS1 |
| -1.286653 | 0.010337 | LAMC2 |
| -1.350239 | 0.042843 | RIPK4 |
| -2.422562 | 0.000655 | CCL20 |
| -1.135308 | 0.021199 | STX1A |
| -1.943847 | 0.000762 | SERPINB2 |
| -1.24316 | 0.008712 | IER3 |
| -1.116196 | 0.034426 | MAML1 |
| -1.4263 | 0.010407 | EGFR |
| -1.148762 | 0.017009 | PXK |
| -1.649913 | 0.001529 | GADD45A |
| -1.035545 | 0.029368 | LYN |
| -1.521494 | 0.007924 | S100A4 |
| -1.122506 | 0.014721 | NFKBIA |
| -1.018316 | 0.027438 | MMP1 |
| -1.157731 | 0.013091 | CCND3 |
| -1.187182 | 0.014536 | LOXL2 |
| -1.516755 | 0.004149 | CEBPB |
| -1.418769 | 0.007363 | LHX6 |
| -1.331626 | 0.009942 | DDIT4 |
| -1.070819 | 0.023125 | MXI1 |
| -1.773488 | 0.005819 | MMP9 |
| -1.615487 | 0.001747 | JDP2 |
| -1.175682 | 0.011872 | MTHFD2 |
| -1.007696 | 0.033274 | MAP2K3 |
| -1.306034 | 0.016192 | SLC1A5 |
| -1.194251 | 0.016782 | SLC7A5 |
| -1.225636 | 0.010315 | BIN1 |
| -1.351505 | 0.006499 | HMOX1 |
| -1.330341 | 0.032349 | ZNF582 |
| -1.140901 | 0.044594 | CASKIN2 |
| -1.667355 | 0.002065 | EIF4EBP2 |
| -1.402803 | 0.007128 | EXOSC5 |
| -1.18611 | 0.011045 | PSAT1 |
| -1.071021 | 0.022564 | ARL14 |
| -1.013406 | 0.025778 | ARHGEF2 |
| -1.038712 | 0.032788 | LIN52 |
| -1.146703 | 0.021623 | JAZF1 |
| -1.674163 | 0.031952 | WDR69 |
| -1.740863 | 0.001424 | ZNF664-FAM101A|FAM101A |
| -1.256491 | 0.020122 | MYEOV |
| -1.118551 | 0.017175 | SFXN1 |
| -1.492179 | 0.003508 | CASP3 |
| -1.409106 | 0.042642 | CAMKV |
| -1.355961 | 0.014178 | FERMT2 |
| -1.281076 | 0.033138 | PRELID2 |
| -1.297837 | 0.007536 | TRIB3 |
| -1.266203 | 0.011673 | CDKN1A |
| -1.17667 | 0.011467 | CCND1 |
| -1.467088 | 0.023281 | ANK3 |
| -1.140944 | 0.012993 | ASNS |
| -1.027255 | 0.023865 | NELF |
| -1.369783 | 0.011536 | CLMP |
| -1.820046 | 0.001009 | EXOSC1 |
| -1.113962 | 0.015773 | SLC3A2 |
| -1.50998 | 0.003218 | GADD45B |
| -1.07099 | 0.037862 | IRAK4 |
| -1.094916 | 0.036836 | PXN |
| -1.043265 | 0.03123 | F8A1 |
| -2.107141 | 0.000223 | EIF4EBP1 |
| -1.171143 | 0.048594 | ARL4C |
| -1.437583 | 0.004736 | S100A2 |
| -1.431928 | 0.008128 | ANKRD20A11P |
| -1.021619 | 0.037422 | LEPREL4 |
| -1.03771 | 0.03771 | FAM211A |
| -1.266228 | 0.008196 | SHMT2 |
| -1.718071 | 0.001143 | CSDAP1|CSDA |
| -1.051129 | 0.048994 | TTLL4 |
| -1.591015 | 0.002094 | CSDA |
| -1.121774 | 0.014734 | DDIT3 |
| -1.237133 | 0.009052 | LYPLA1 |
| -1.979708 | 0.002194 | NINJ1 |
| -1.624752 | 0.020123 | GDF15 |
| -2.05392 | 0.001345 | TNFRSF9 |
| -1.156695 | 0.032966 | TWIST2 |
| -1.173507 | 0.012653 | SNHG1 |
| -1.497338 | 0.01228 | LOC100129034 |
| -1.006814 | 0.029079 | DNAJB6 |
| -1.109409 | 0.046344 | GPCPD1 |
| -1.170602 | 0.026866 | RFK |
| -1.421139 | 0.032725 | OLFML3 |
| -1.852926 | 0.000652 | OR4B1 |
| -2.027288 | 0.022333 | ULBP1 |
| -1.949744 | 0.001228 | CXCL2 |
| -1.540053 | 0.006335 | BNIP3L |
| -1.212693 | 0.01635 | CITED1 |
| -1.012036 | 0.047817 | CALB2 |
| -1.327801 | 0.013215 | LAMC2 |
| -1.371233 | 0.00697 | CSRP2 |
| -1.241903 | 0.020808 | NA |
| -1.099902 | 0.017126 | HIPK2 |
| -1.027052 | 0.033205 | SFXN1 |
| -1.581873 | 0.002618 | RMND5A |
| -1.480907 | 0.01242 | CCDC68 |
| -1.355476 | 0.010959 | SLC7A11 |
| -1.021797 | 0.046978 | PIK3R3 |
| -1.293267 | 0.021699 | USP36 |
| -1.360131 | 0.004821 | DNAJB6 |
| -1.007729 | 0.049124 | SLX1B|SLX1A |

1. **Fold difference (log2 (Ratio of miR-138/control)) >1.00 (up-regulated) or <-1.00 (down-regulated), and p-value < 0.05.**

**Supplementary Table2：Mass spectrometry analysis results of the potential protein targets of RMND5A interaction （Protein scores of Mascot Search Results >100）.**

| **Anti-Myc** | **NCBInr accession No** | **Mass** | **Score** | **Peptides matched** | **Protein name** |
| --- | --- | --- | --- | --- | --- |
| 1 | gi| 28839692 | 43993 | 2015 | 40（39） | required for meiotic nuclear division 5 homolog A |
| 2 | gi|15080674 | 77847 | 1702 | 38(31) | Ran Binding Protein in the Microtubule organizing center |
| 3 | gi|22748937 | 窗体顶端  136222窗体底端 | 1317 | 37(27) | 窗体顶端  exportin-5 窗体底端 |
| 4 | gi|31621305 | 157805 | 956 | 27(17) | leucine-rich PPR motif-containing protein, mitochondrial precursor |
| 5 | gi|153792590 | 98099 | 947 | 38(21) | heat shock protein HSP 90-alpha isoform 1 |
| 6 | gi|4507877 | 116649 | 810 | 23(14) | vinculin isoform VCL |
| 7 | gi|35830 | 117715 | 702 | 18(16) | ubiquitin activating enzyme E1 |
| 8 | gi|21626466 | 94565 | 520 | 14(8) | matrin-3 isoform a |
| 9 | gi|61744477 | 68059 | 464 | 10(8) | 4F2 cell-surface antigen heavy chain isoform b |
| 10 | gi|32358 | 88890 | 393 | 10(7) | hnRNP U protein |
| 11 | gi|18700635 | 118642 | 329 | 10(6) | Importin-4 |
| 12 | gi|4503483 | 95277 | 317 | 10(6) | elongation factor 2 |
| 13 | gi|38327039 | 94271 | 301 | 10(6) | heat shock 70 kDa protein 4 |
| 14 | gi|61104911 | 49092 | 292 | 10(5) | heat shock protein 90Bb |
| 15 | gi|119629383 | 窗体顶端  125507  窗体底端 | 窗体顶端  249  窗体底端 | 窗体顶端  9(6)  窗体底端 | 窗体顶端  RAN binding protein 5, isoform CRA_b窗体底端 |
| 16 | gi|11544639 | 窗体顶端  116304窗体底端 | 窗体顶端  228窗体底端 | 窗体顶端  11(4)窗体底端 | Importin-7 |
| 17 | gi|4507943 | 窗体顶端  123306窗体底端 | 窗体顶端  228窗体底端 | 窗体顶端  3(3)窗体底端 | 窗体顶端  exportin-1 窗体底端 |
| 18 | gi|3170190 | 窗体顶端  98434窗体底端 | 窗体顶端  217窗体底端 | 窗体顶端  9(5)窗体底端 | 窗体顶端  antigen NY-CO-25窗体底端 |
| 19 | gi|4503471 | 窗体顶端  50109窗体底端 | 窗体顶端  152窗体底端 | 窗体顶端  8(3)窗体底端 | 窗体顶端  elongation factor 1-alpha 1窗体底端 |
| 20 | gi|603074 | 窗体顶端  120748窗体底端 | 150 | 窗体顶端  8(3)窗体底端 | 窗体顶端  ATP:citrate lyase 窗体底端 |
| 21 | gi|190167 | 窗体顶端  113011窗体底端 | 窗体顶端  148窗体底端 | 窗体顶端  6(1)窗体底端 | 窗体顶端  poly(ADP-ribose) polymerase窗体底端 |
| 22 | gi|4505257 | 窗体顶端  67778窗体底端 | 窗体顶端  139窗体底端 | 窗体顶端  8(4)窗体底端 | 窗体顶端  moesin窗体底端 |
| 23 | gi|5453607 | 窗体顶端  59329  窗体底端 | 窗体顶端  128  窗体底端 | 窗体顶端  1(1)  窗体底端 | 窗体顶端  T-complex protein 1 subunit eta isoform a |
| 24 | gi|458032 | 窗体顶端  85445窗体底端 | 窗体顶端  124窗体底端 | 窗体顶端  2(1)窗体底端 | 窗体顶端  aspartyl beta-hydroxylase窗体底端 |
| 25 | gi|1911652 | 窗体顶端  85837窗体底端 | 窗体顶端  123窗体底端 | 窗体顶端  3(1)  窗体底端 | 窗体顶端  aspartyl(asparaginyl)beta-hydroxylase窗体底端 |
| 26 | gi|5729877 | 70854 | 121 | 窗体顶端  5(2)窗体底端 | 窗体顶端  heat shock cognate 71 kDa protein isoform 1窗体底端 |
| 27 | gi|19743813 | 88357 | 120 | 4(2) | integrin beta-1 isoform 1A precursor |
| 28 | gi|459214638 | 155718 | 112 | 4(1) | 窗体顶端  KIF5B-RET(NM_020630)_K24;R11 fusion protein窗体底端 |

**Supplementary Table3：Differentially regulated microRNAs by miR-138^a^ in HeLa cells**

| miRNAs Name | p-value | A-miHeLa-NC-Af5 | | B-miHeLa-138-Af3 | log2 (B/A) |
| --- | --- | --- | --- | --- | --- |
| hsa-miR-4281 | 3.41E-03 | 2,376 | | 6,146 | 1.37 |
| hsa-miR-138 | 8.80E-03 | 125 | | 14,773 | 6.89 |
| hsa-miR-217 | 9.67E-03 | 16 | | 15,194 | 9.93 |
| hsa-miR-4497 | 1.01E-02 | 3,075 | | 5,290 | 0.78 |
| hsa-miR-3960 | 1.10E-02 | 19,658 | | 27,055 | 0.46 |
| hsa-miR-125b | 1.13E-02 | 1,705 | | 372 | -2.20 |
| hsa-miR-3665 | 1.60E-02 | 13,128 | | 30,090 | 1.20 |
| hsa-miR-4668-5p | 1.64E-02 | 1,208 | | 8,937 | 2.89 |
| hsa-miR-17 | 1.66E-02 | 4,427 | | 1,410 | -1.65 |
| hsa-miR-4530 | 1.78E-02 | 1,614 | | 5,783 | 1.84 |
| hsa-miR-30a | 1.93E-02 | 1,330 | | 342 | -1.96 |
| hsa-miR-20a | 2.04E-02 | 4,498 | | 1,422 | -1.66 |
| hsa-miR-188-5p | 2.24E-02 | 262 | | 803 | 1.62 |
| hsa-miR-4687-3p | 2.35E-02 | 2,460 | | 5,354 | 1.12 |
| hsa-miR-23b | 2.41E-02 | 12,426 | | 3,740 | -1.73 |
| hsa-miR-30d | 2.68E-02 | 591 | | 143 | -2.05 |
| hsa-miR-138-1* | 2.79E-02 | 147 | | 508 | 1.79 |
| hsa-miR-4454 | 2.80E-02 | 5,849 | | 2,064 | -1.50 |
| hsa-miR-4534 | 3.04E-02 | 1,169 | | 2,293 | 0.97 |
| hsa-miR-106a | 3.15E-02 | 3,948 | | 1,234 | -1.68 |
| hsa-miR-22 | 3.23E-02 | 529 | | 157 | -1.75 |
| hsa-miR-26a | 3.23E-02 | 1,669 | | 526 | -1.67 |
| hsa-miR-320b | 3.24E-02 | 1,543 | | 716 | -1.11 |
| hsa-miR-151-5p | 3.48E-02 | 1,238 | | 462 | -1.42 |
| hsa-miR-1224-5p | 3.61E-02 | 242 | | 718 | 1.57 |
| hsa-miR-16 | 3.65E-02 | 3,859 | | 1,342 | -1.52 |
| hsa-miR-107 | 3.82E-02 | 1,184 | | 391 | -1.60 |
| hsa-miR-1915 | 3.83E-02 | 901 | | 2,169 | 1.27 |
| hsa-miR-23a | 3.94E-02 | 14,434 | | 4,918 | -1.55 |
| hsa-miR-320c | 3.99E-02 | 1,957 | | 816 | -1.26 |
| hsa-miR-4499 | 4.02E-02 | 855 | | 1,885 | 1.14 |
| hsa-miR-99a | 4.07E-02 | 541 | | 71 | -2.94 |
| hsa-miR-3656 | 4.13E-02 | 2,574 | | 6,358 | 1.30 |
| hsa-miR-4787-5p | 4.15E-02 | 7,067 | | 10,430 | 0.56 |
| hsa-miR-2861 | 4.16E-02 | 452 | | 1,972 | 2.13 |
| hsa-miR-221 | 4.22E-02 | 2,550 | | 682 | -1.90 |
| hsa-miR-320a | 4.91E-02 | 2,127 | | 958 | -1.15 |
| hsa-miR-4707-5p | 4.92E-02 | 1,130 | | 2,726 | 1.27 |
| hsa-miR-106b | 4.95E-02 | 808 | | 384 | -1.07 |
| hsa-miR-149* | 5.15E-02 | 182 | | 708 | 1.96 |
| hsa-miR-4298 | 5.26E-02 | 4,498 | | 8,601 | 0.93 |
| hsa-miR-1268 | 5.33E-02 | 760 | | 1,711 | 1.17 |
| hsa-miR-1280 | 5.46E-02 | 2,888 | | 1,238 | -1.22 |
| hsa-miR-4466 | 5.47E-02 | 1,438 | | 3,018 | 1.07 |
| hsa-miR-1469 | 5.60E-02 | 105 | | 555 | 2.40 |
| hsa-miR-762 | 5.66E-02 | 338 | | 1,062 | 1.65 |
| hsa-miR-224 | 5.67E-02 | 857 | | 355 | -1.27 |
| hsa-miR-4484 | 5.89E-02 | 3,405 | | 4,769 | 0.49 |
| hsa-miR-574-3p | 5.93E-02 | 7,062 | | 5,625 | -0.33 |
| hsa-miR-638 | 5.96E-02 | 3,400 | | 6,704 | 0.98 |
| hsa-miR-4739 | 6.30E-02 | 3,276 | | 5,744 | 0.81 |
| hsa-let-7d | 6.43E-02 | 4,460 | | 1,664 | -1.42 |
| hsa-miR-3940-5p | 6.49E-02 | 848 | | 1,822 | 1.10 |
| hsa-miR-720 | 6.52E-02 | 1,539 | | 723 | -1.09 |
| hsa-miR-1268b | 6.62E-02 | 801 | | 1,712 | 1.10 |
| hsa-miR-4443 | 6.63E-02 | 5,713 | | 3,729 | -0.62 |
| hsa-miR-30c | 6.64E-02 | 2,919 | | 678 | -2.11 |
| hsa-miR-194 | 6.78E-02 | 501 | | 199 | -1.33 |
| hsa-miR-3141 | 6.90E-02 | 4,438 | | 10,120 | 1.19 |
| hsa-miR-3610 | 7.11E-02 | 415 | | 1,001 | 1.27 |
| hsa-miR-320d | 7.14E-02 | 1,122 | | 295 | -1.93 |
| hsa-miR-20b | 7.31E-02 | 1,120 | | 388 | -1.53 |
| hsa-miR-27b | 1.36E-01 | 1,550 | | 690 | -1.17 |
| hsa-miR-15b | 7.68E-02 | 1,988 | | 670 | -1.57 |
| hsa-miR-4516 | 7.70E-02 | 4,071 | | 7,724 | 0.92 |
| hsa-let-7f | 7.93E-02 | 6,509 | | 2,368 | -1.46 |
| hsa-miR-4521 | 7.94E-02 | 514 | | 227 | -1.18 |
| hsa-miR-29a | 8.02E-02 | 1,838 | | 398 | -2.21 |
| hsa-miR-4442 | 8.03E-02 | 915 | | 2,394 | 1.39 |
| hsa-miR-92a | 8.04E-02 | 9,810 | | 4,620 | -1.09 |
| hsa-miR-671-5p | 8.13E-02 | 240 | | 606 | 1.34 |
| hsa-miR-361-5p | 8.24E-02 | 758 | | 333 | -1.19 |
| hsa-miR-27a | 8.38E-02 | 4,002 | | 1,423 | -1.49 |
| hsa-miR-151b | 8.96E-02 | 1,094 | | 413 | -1.41 |
| hsa-miR-222 | 9.07E-02 | 3,706 | | 2,078 | -0.83 |
| hsa-miR-182 | 9.39E-02 | 506 | | 200 | -1.34 |
| hsa-miR-30b | 9.78E-02 | 1,412 | | 337 | -2.07 |
| hsa-miR-92b | 9.79E-02 | 3,482 | | 1,264 | -1.46 |
| hsa-miR-940 | 9.92E-02 | 165 | | 637 | 1.95 |
| hsa-miR-31 | 9.94E-02 | 2,982 | | 1,224 | -1.29 |
| Following transcripts are statistically significant but have low signals (signal < 500) | | | | | |
| hsa-miR-142 | 4.69E-03 | 258 | | 51 | -2.34 |
| hsa-miR-181a | 2.75E-02 | 431 | | 139 | -1.63 |
| hsa-miR-584 | 2.87E-02 | 142 | | 40 | -1.82 |
| hsa-miR-1249 | 3.04E-02 | 37 | | 101 | 1.43 |
| hsa-miR-3609 | 3.39E-02 | 170 | | 54 | -1.66 |
| hsa-miR-3667-5p | 3.42E-02 | 114 | | 252 | 1.15 |
| hsa-miR-425 | 3.44E-02 | 237 | | 39 | -2.61 |
| hsa-miR-128 | 3.54E-02 | 271 | | 81 | -1.75 |
| hsa-miR-1238 | 3.84E-02 | 25 | | 77 | 1.60 |
| hsa-miR-140-5p | 4.09E-02 | 22 | | 0 | -14.48 |
| hsa-miR-654-3p | 4.44E-02 | 147 | | 68 | -1.11 |
| hsa-miR-4764-3p | 4.46E-02 | 32 | | 19 | -0.76 |
| hsa-miR-151-3p | 4.81E-02 | 358 | | 133 | -1.43 |
| hsa-miR-203 | 5.04E-02 | 66 | | 13 | -2.37 |
| hsa-miR-196a | 5.23E-02 | 289 | | 73 | -1.99 |
| hsa-miR-3180 | 5.30E-02 | 20 | | 47 | 1.26 |
| hsa-miR-3131 | 5.38E-02 | 54 | | 101 | 0.91 |
| hsa-miR-4270 | 5.39E-02 | 190 | | 464 | 1.29 |
| hsa-miR-296-5p | 5.65E-02 | 37 | | 86 | 1.20 |
| hsa-miR-665 | 5.67E-02 | 45 | | 72 | 0.66 |
| hsa-miR-192 | 5.70E-02 | 77 | | 29 | -1.38 |
| hsa-miR-603 | 5.90E-02 | 47 | | 7 | -2.78 |
| hsa-miR-4535 | 6.27E-02 | 62 | | 108 | 0.79 |
| hsa-miR-3180-3p | 6.29E-02 | 20 | | 34 | 0.81 |
| hsa-miR-200c | 6.36E-02 | 104 | | 17 | -2.60 |
| hsa-miR-3181 | 6.38E-02 | 17 | | 38 | 1.15 |
| hsa-miR-629 | 6.52E-02 | 101 | | 29 | -1.80 |
| hsa-miR-30e | 6.55E-01 | 204 | | 17 | -3.58 |
| hsa-miR-744 | 6.56E-02 | 70 | | 24 | -1.55 |
| hsa-miR-548q | 6.62E-02 | 38 | | 99 | 1.38 |
| hsa-miR-25* | 6.62E-02 | 105 | | 24 | -2.10 |
| hsa-miR-150* | 6.62E-02 | 70 | | 133 | 0.93 |
| hsa-miR-3127-5p | 6.66E-02 | 73 | | 33 | -1.13 |
| hsa-miR-502-3p | 6.67E-02 | 53 | | 21 | -1.32 |
| hsa-miR-130b | 6.96E-02 | 136 | | 43 | -1.66 |
| hsa-miR-4278 | 7.19E-02 | 29 | | 55 | 0.94 |
| hsa-miR-4429 | 7.76E-02 | 192 | | 73 | -1.40 |
| hsa-miR-134 | 8.20E-02 | 65 | | 181 | 1.48 |
| hsa-miR-30c-2* | 8.34E-02 | 37 | | 17 | -1.09 |
| hsa-miR-331-3p | 8.66E-02 | 113 | | 58 | -0.95 |
| hsa-miR-23c | 8.72E-02 | 156 | | 51 | -1.61 |
| hsa-miR-423-5p | 8.76E-02 | 480 | | 357 | -0.43 |
| hsa-miR-3194-5p | 8.78E-02 | 31 | | 61 | 0.99 |
| hsa-miR-4324 | 8.79E-02 | 113 | | 18 | -2.62 |
| hsa-miR-99b | 8.79E-02 | 355 | | 117 | -1.61 |
| hsa-miR-7 | 8.88E-02 | 362 | | 52 | -2.79 |
| hsa-miR-3663-5p | 9.03E-02 | 30 | | 44 | 0.55 |
| hsa-miR-125a-3p | 9.41E-02 | 59 | | 93 | 0.66 |
| hsa-miR-3185 | 9.47E-02 | 97 | | 174 | 0.85 |
| hsa-miR-625 | 9.53E-02 | 72 | | 21 | -1.81 |
| hsa-miR-4327 | 9.71E-02 | 37 | | 94 | 1.35 |
| hsa-miR-371-5p | 9.73E-02 | 50 | | 154 | 1.61 |
| hsa-miR-3679-5p | 9.78E-02 | | 28 | 47 | 0.75 |
| Following transcripts are statistically insignificant (p-value >0.1) | | | | | |
| hsa-miR-595 | 1.03E-01 | 134 | | 95 | -0.49 |
| hsa-miR-765 | 1.03E-01 | 350 | | 753 | 1.11 |
| hsa-miR-371b-5p | 1.03E-01 | 169 | | 303 | 0.84 |
| hsa-miR-202 | 1.04E-01 | 36 | | 21 | -0.77 |
| hsa-miR-3713 | 1.06E-01 | 38 | | 66 | 0.80 |
| hsa-miR-103a | 1.07E-01 | 1,163 | | 412 | -1.50 |
| hsa-let-7g | 1.07E-01 | 2,061 | | 584 | -1.82 |
| hsa-miR-617 | 1.08E-01 | 132 | | 285 | 1.11 |
| hsa-miR-342-3p | 1.08E-01 | 120 | | 48 | -1.33 |
| hsa-miR-3652 | 1.08E-01 | 86 | | 159 | 0.89 |
| hsa-miR-4743 | 1.08E-01 | 226 | | 355 | 0.66 |
| hsa-miR-1307 | 1.08E-01 | 205 | | 126 | -0.71 |
| hsa-miR-4419b | 1.09E-01 | 310 | | 749 | 1.27 |
| hsa-miR-30e* | 1.09E-01 | 59 | | 13 | -2.23 |
| hsa-miR-576-3p | 1.11E-01 | 33 | | 1 | -5.11 |
| hsa-miR-4444 | 1.12E-01 | 241 | | 650 | 1.43 |
| hsa-miR-601 | 1.12E-01 | 566 | | 920 | 0.70 |
| hsa-miR-494 | 1.12E-01 | 117 | | 437 | 1.91 |
| hsa-miR-3655 | 1.13E-01 | 22 | | 34 | 0.65 |
| hsa-miR-451b | 1.14E-01 | 32 | | 10 | -1.60 |
| hsa-miR-4655-3p | 1.15E-01 | 15 | | 67 | 2.16 |
| hsa-miR-454 | 1.16E-01 | 56 | | 24 | -1.24 |
| hsa-miR-27b* | 1.18E-01 | 73 | | 23 | -1.66 |
| hsa-miR-10a | 1.18E-01 | 50 | | 16 | -1.65 |
| hsa-miR-3659 | 1.18E-01 | 18 | | 41 | 1.16 |
| hsa-miR-124 | 1.19E-01 | 39 | | 17 | -1.20 |
| hsa-miR-575 | 1.20E-01 | 71 | | 195 | 1.45 |
| hsa-miR-4637 | 1.21E-01 | 56 | | 15 | -1.94 |
| hsa-miR-4769-5p | 1.21E-01 | 4 | | 35 | 2.99 |
| hsa-miR-3613-3p | 1.22E-01 | 213 | | 6,630 | 4.96 |
| hsa-miR-183* | 1.24E-01 | 29 | | 5 | -2.49 |
| hsa-miR-320e | 1.25E-01 | 814 | | 243 | -1.74 |
| hsa-miR-4488 | 1.28E-01 | 3,692 | | 4,672 | 0.34 |
| hsa-miR-4649-5p | 1.28E-01 | 37 | | 72 | 0.97 |
| hsa-miR-4505 | 1.28E-01 | 406 | | 1,031 | 1.34 |
| hsa-miR-23a* | 1.29E-01 | 181 | | 78 | -1.22 |
| hsa-miR-191 | 1.30E-01 | 1,340 | | 454 | -1.56 |
| hsa-miR-631 | 1.30E-01 | 94 | | 50 | -0.92 |
| hsa-miR-500a* | 1.31E-01 | 85 | | 26 | -1.72 |
| hsa-miR-4741 | 1.31E-01 | 65 | | 181 | 1.49 |
| hsa-miR-568 | 1.31E-01 | 425 | | 1,141 | 1.42 |
| hsa-miR-125a-5p | 1.31E-01 | 286 | | 78 | -1.88 |
| hsa-miR-4778-5p | 1.32E-01 | 3,956 | | 8,175 | 1.05 |
| hsa-miR-1247 | 1.33E-01 | 17 | | 32 | 0.89 |
| hsa-miR-4508 | 1.34E-01 | 1,360 | | 2,480 | 0.87 |
| hsa-miR-25 | 1.35E-01 | 2,826 | | 1,197 | -1.24 |
| hsa-miR-483-5p | 1.36E-01 | 1,509 | | 2,572 | 0.77 |
| hsa-miR-4750 | 1.37E-01 | 61 | | 69 | 0.18 |
| hsa-miR-135a* | 1.37E-01 | 27 | | 53 | 0.96 |
| hsa-miR-484 | 1.37E-01 | 138 | | 90 | -0.61 |
| hsa-miR-663 | 1.38E-01 | 791 | | 861 | 0.12 |
| hsa-miR-4463 | 1.38E-01 | 585 | | 1,293 | 1.14 |
| hsa-miR-4638-3p | 1.39E-01 | 78 | | 39 | -0.99 |
| hsa-miR-3667-3p | 1.40E-01 | 14 | | 32 | 1.22 |
| hsa-miR-1290 | 1.41E-01 | 50 | | 139 | 1.46 |
| hsa-miR-622 | 1.43E-01 | 71 | | 38 | -0.89 |
| hsa-miR-3180-5p | 1.43E-01 | 33 | | 75 | 1.17 |
| hsa-miR-138-2* | 1.44E-01 | 29 | | 10 | -1.60 |
| hsa-miR-3147 | 1.44E-01 | 91 | | 141 | 0.62 |
| hsa-miR-3135b | 1.45E-01 | 34 | | 62 | 0.88 |
| hsa-miR-532-5p | 1.45E-01 | 95 | | 35 | -1.44 |
| hsa-miR-100 | 1.46E-01 | 687 | | 227 | -1.60 |
| hsa-miR-18a | 1.46E-01 | 159 | | 39 | -2.05 |
| hsa-miR-183 | 1.48E-01 | 127 | | 36 | -1.82 |
| hsa-miR-766 | 1.48E-01 | 93 | | 45 | -1.07 |
| hsa-miR-4417 | 1.49E-01 | 26 | | 40 | 0.62 |
| hsa-miR-4732-5p | 1.50E-01 | 198 | | 481 | 1.28 |
| hsa-miR-1260b | 1.50E-01 | 764 | | 299 | -1.35 |
| hsa-miR-206 | 1.51E-01 | 70 | | 43 | -0.72 |
| hsa-miR-4690-5p | 1.52E-01 | 115 | | 234 | 1.03 |
| hsa-miR-4423-3p | 1.52E-01 | 315 | | 209 | -0.59 |
| hsa-miR-24-2* | 1.56E-01 | 91 | | 11 | -3.03 |
| hsa-miR-1908 | 1.57E-01 | 52 | | 93 | 0.84 |
| hsa-let-7i | 1.59E-01 | 2,034 | | 892 | -1.19 |
| hsa-miR-3124-5p | 1.61E-01 | 27 | | 62 | 1.22 |
| hsa-miR-3196 | 1.63E-01 | 1,796 | | 2,570 | 0.52 |
| hsa-let-7c | 1.63E-01 | 4,354 | | 2,008 | -1.12 |
| hsa-miR-629* | 1.67E-01 | 89 | | 51 | -0.82 |
| hsa-miR-193b | 1.67E-01 | 89 | | 42 | -1.07 |
| hsa-miR-4652-3p | 1.69E-01 | 7 | | 34 | 2.36 |
| hsa-miR-5096 | 1.69E-01 | 1,300 | | 1,766 | 0.44 |
| hsa-miR-24 | 1.70E-01 | 4,426 | | 2,703 | -0.71 |
| hsa-miR-216a | 1.71E-01 | 30 | | 17 | -0.82 |
| hsa-miR-186 | 1.71E-01 | 64 | | 15 | -2.14 |
| hsa-miR-3666 | 1.71E-01 | 15 | | 29 | 0.96 |
| hsa-miR-505* | 1.72E-01 | 40 | | 15 | -1.38 |
| hsa-miR-3183 | 1.73E-01 | 29 | | 20 | -0.56 |
| hsa-miR-642b | 1.78E-01 | 96 | | 224 | 1.23 |
| hsa-let-7b | 1.80E-01 | 2,854 | | 1,532 | -0.90 |
| hsa-miR-1281 | 1.81E-01 | 78 | | 316 | 2.03 |
| hsa-miR-3654 | 1.81E-01 | 107 | | 58 | -0.89 |
| hsa-miR-4644 | 1.83E-01 | 44 | | 12 | -1.90 |
| hsa-miR-4684-3p | 1.84E-01 | 75 | | 37 | -1.02 |
| hsa-miR-654-5p | 1.85E-01 | 82 | | 137 | 0.74 |
| hsa-miR-4290 | 1.85E-01 | 56 | | 119 | 1.09 |
| hsa-miR-4734 | 1.87E-01 | 1,381 | | 2,545 | 0.88 |
| hsa-miR-3149 | 1.87E-01 | 615 | | 813 | 0.40 |
| hsa-miR-3679-3p | 1.88E-01 | 34 | | 53 | 0.65 |
| hsa-miR-3187-3p | 1.89E-01 | 28 | | 65 | 1.25 |
| hsa-miR-2392 | 1.89E-01 | 93 | | 168 | 0.86 |
| hsa-miR-32* | 1.89E-01 | 81 | | 34 | -1.25 |
| hsa-miR-663b | 1.90E-01 | 26 | | 35 | 0.43 |
| hsa-miR-4655-5p | 1.90E-01 | 60 | | 104 | 0.80 |
| hsa-miR-2355-5p | 1.90E-01 | 32 | | 7 | -2.16 |
| hsa-miR-324-5p | 1.92E-01 | 105 | | 44 | -1.27 |
| hsa-miR-4507 | 1.93E-01 | 248 | | 519 | 1.07 |
| hsa-miR-185 | 1.94E-01 | 220 | | 78 | -1.49 |
| hsa-miR-4459 | 1.94E-01 | 7,463 | | 12,463 | 0.74 |
| hsa-miR-373* | 1.97E-01 | 18 | | 44 | 1.28 |
| hsa-miR-4419a | 1.98E-01 | 108 | | 149 | 0.47 |
| hsa-miR-181b | 2.00E-01 | 192 | | 116 | -0.73 |
| hsa-miR-4783-3p | 2.01E-01 | 28 | | 64 | 1.19 |
| hsa-miR-197 | 2.01E-01 | 124 | | 70 | -0.83 |
| hsa-miR-4656 | 2.02E-01 | 84 | | 131 | 0.64 |
| hsa-miR-132 | 2.02E-01 | 86 | | 33 | -1.39 |
| hsa-miR-3176 | 2.03E-01 | 15 | | 35 | 1.22 |
| hsa-miR-4654 | 2.03E-01 | 7 | | 25 | 1.74 |
| hsa-miR-615-3p | 2.04E-01 | 48 | | 30 | -0.72 |
| hsa-miR-503 | 2.04E-01 | 58 | | 19 | -1.61 |
| hsa-miR-1228* | 2.06E-01 | 229 | | 360 | 0.65 |
| hsa-miR-501-5p | 2.07E-01 | 34 | | 17 | -0.97 |
| hsa-miR-3130-3p | 2.07E-01 | 26 | | 31 | 0.26 |
| hsa-miR-4513 | 2.07E-01 | 64 | | 146 | 1.20 |
| hsa-miR-139-5p | 2.10E-01 | 30 | | 12 | -1.30 |
| hsa-miR-30a* | 2.12E-01 | 214 | | 78 | -1.46 |
| hsa-let-7a | 2.13E-01 | 7,967 | | 4,302 | -0.89 |
| hsa-miR-4787-3p | 2.14E-01 | 33 | | 57 | 0.81 |
| hsa-miR-500b | 2.15E-01 | 36 | | 28 | -0.35 |
| hsa-miR-210 | 2.15E-01 | 127 | | 75 | -0.76 |
| hsa-miR-103a-2* | 2.16E-01 | 33 | | 19 | -0.83 |
| hsa-miR-744* | 2.20E-01 | 45 | | 11 | -1.99 |
| hsa-miR-659 | 2.22E-01 | 41 | | 52 | 0.35 |
| hsa-miR-625* | 2.23E-01 | 42 | | 56 | 0.43 |
| hsa-miR-4731-3p | 2.23E-01 | 41 | | 84 | 1.04 |
| hsa-miR-1825 | 2.23E-01 | 53 | | 80 | 0.58 |
| hsa-miR-4299 | 2.27E-01 | 25 | | 55 | 1.15 |
| hsa-miR-1913 | 2.27E-01 | 54 | | 97 | 0.85 |
| hsa-miR-1275 | 2.28E-01 | 697 | | 1,059 | 0.60 |
| hsa-miR-4763-3p | 2.29E-01 | 101 | | 68 | -0.58 |
| hsa-miR-4274 | 2.29E-01 | 26 | | 100 | 1.95 |
| hsa-miR-4292 | 2.29E-01 | 18 | | 51 | 1.48 |
| hsa-miR-921 | 2.30E-01 | 357 | | 198 | -0.85 |
| hsa-miR-4254 | 2.30E-01 | 152 | | 40 | -1.93 |
| hsa-miR-3137 | 2.31E-01 | 20 | | 34 | 0.79 |
| hsa-miR-4748 | 2.31E-01 | 26 | | 33 | 0.36 |
| hsa-miR-195 | 2.32E-01 | 72 | | 40 | -0.86 |
| hsa-miR-4770 | 2.32E-01 | 4 | | 24 | 2.69 |
| hsa-miR-4776-5p | 2.33E-01 | 20 | | 38 | 0.91 |
| hsa-miR-3689a-3p | 2.34E-01 | 4 | | 24 | 2.45 |
| hsa-miR-4492 | 2.34E-01 | 363 | | 492 | 0.44 |
| hsa-miR-21 | 2.34E-01 | 25,531 | | 19,938 | -0.36 |
| hsa-miR-3714 | 2.34E-01 | 25 | | 51 | 1.02 |
| hsa-miR-424 | 2.35E-01 | 39 | | 6 | -2.82 |
| hsa-miR-1183 | 2.35E-01 | 100 | | 66 | -0.60 |
| hsa-miR-7-1* | 2.38E-01 | 41 | | 8 | -2.31 |
| hsa-miR-4269 | 2.39E-01 | 110 | | 50 | -1.13 |
| hsa-miR-4280 | 2.40E-01 | 25 | | 49 | 1.01 |
| hsa-miR-26b | 2.41E-01 | 232 | | 87 | -1.42 |
| hsa-miR-4312 | 2.42E-01 | 19 | | 38 | 1.00 |
| hsa-miR-3122 | 2.43E-01 | 17 | | 36 | 1.10 |
| hsa-miR-1207-5p | 2.43E-01 | 76 | | 157 | 1.05 |
| hsa-miR-668 | 2.44E-01 | 29 | | 20 | -0.55 |
| hsa-miR-93 | 2.44E-01 | 1,435 | | 1,068 | -0.43 |
| hsa-miR-1234 | 2.45E-01 | 46 | | 108 | 1.24 |
| hsa-miR-4478 | 2.46E-01 | 125 | | 218 | 0.80 |
| hsa-miR-4306 | 2.46E-01 | 120 | | 65 | -0.88 |
| hsa-miR-3676 | 2.47E-01 | 102 | | 140 | 0.46 |
| hsa-miR-4747-3p | 2.49E-01 | 27 | | 34 | 0.32 |
| hsa-miR-4421 | 2.50E-01 | 28 | | 17 | -0.71 |
| hsa-miR-4252 | 2.52E-01 | 51 | | 21 | -1.29 |
| hsa-miR-3663-3p | 2.52E-01 | 253 | | 202 | -0.32 |
| hsa-miR-4291 | 2.53E-01 | 24 | | 46 | 0.91 |
| hsa-miR-196b* | 2.55E-01 | 81 | | 54 | -0.59 |
| hsa-miR-18b | 2.55E-01 | 29 | | 6 | -2.17 |
| hsa-miR-609 | 2.56E-01 | 49 | | 28 | -0.79 |
| hsa-miR-4776-3p | 2.56E-01 | 10 | | 26 | 1.44 |
| hsa-miR-4433 | 2.58E-01 | 171 | | 275 | 0.69 |
| hsa-miR-378 | 2.60E-01 | 186 | | 98 | -0.92 |
| hsa-miR-3648 | 2.60E-01 | 24 | | 50 | 1.06 |
| hsa-miR-3162-3p | 2.61E-01 | 18 | | 80 | 2.11 |
| hsa-miR-16-2* | 2.62E-01 | 57 | | 16 | -1.85 |
| hsa-miR-181c | 2.64E-01 | 52 | | 17 | -1.57 |
| hsa-miR-1254 | 2.64E-01 | 25 | | 36 | 0.49 |
| hsa-miR-23b* | 2.66E-01 | 28 | | 10 | -1.54 |
| hsa-miR-636 | 2.67E-01 | 29 | | 72 | 1.31 |
| hsa-miR-670 | 2.69E-01 | 73 | | 51 | -0.52 |
| hsa-miR-3197 | 2.69E-01 | 77 | | 38 | -1.04 |
| hsa-miR-3935 | 2.72E-01 | 122 | | 185 | 0.60 |
| hsa-miR-374b | 2.72E-01 | 66 | | 31 | -1.10 |
| hsa-miR-365 | 2.73E-01 | 67 | | 41 | -0.72 |
| hsa-miR-224* | 2.75E-01 | 43 | | 18 | -1.25 |
| hsa-miR-4730 | 2.75E-01 | 68 | | 30 | -1.18 |
| hsa-miR-3178 | 2.79E-01 | 550 | | 643 | 0.23 |
| hsa-miR-4447 | 2.79E-01 | 54 | | 113 | 1.07 |
| hsa-miR-4663 | 2.81E-01 | 19 | | 32 | 0.75 |
| hsa-miR-1587 | 2.81E-01 | 231 | | 401 | 0.80 |
| hsa-miR-4745-5p | 2.81E-01 | 33 | | 50 | 0.58 |
| hsa-miR-874 | 2.82E-01 | 22 | | 78 | 1.83 |
| hsa-miR-1267 | 2.82E-01 | 13 | | 45 | 1.83 |
| hsa-miR-3175 | 2.83E-01 | 13 | | 30 | 1.28 |
| hsa-miR-204 | 2.83E-01 | 31 | | 0 | -6.32 |
| hsa-miR-200b* | 2.85E-01 | 33 | | 12 | -1.49 |
| hsa-miR-877* | 2.85E-01 | 22 | | 57 | 1.35 |
| hsa-miR-365* | 2.86E-01 | 16 | | 37 | 1.20 |
| hsa-miR-3177-3p | 2.87E-01 | 43 | | 67 | 0.63 |
| hsa-miR-4667-5p | 2.88E-01 | 149 | | 228 | 0.61 |
| hsa-miR-4257 | 2.91E-01 | 33 | | 64 | 0.98 |
| hsa-miR-3607-5p | 2.92E-01 | 38 | | 17 | -1.20 |
| hsa-miR-1181 | 2.94E-01 | 36 | | 51 | 0.52 |
| hsa-miR-3139 | 2.95E-01 | 13 | | 27 | 1.05 |
| hsa-miR-4642 | 2.96E-01 | 33 | | 21 | -0.66 |
| hsa-miR-4634 | 2.96E-01 | 30 | | 46 | 0.61 |
| hsa-miR-3622a-5p | 2.97E-01 | 4 | | 23 | 2.61 |
| hsa-miR-4792 | 2.98E-01 | 18 | | 36 | 1.01 |
| hsa-miR-3621 | 2.99E-01 | 29 | | 101 | 1.82 |
| hsa-miR-4294 | 2.99E-01 | 38 | | 69 | 0.85 |
| hsa-miR-937 | 3.00E-01 | 21 | | 36 | 0.76 |
| hsa-miR-3192 | 3.00E-01 | 16 | | 26 | 0.74 |
| hsa-miR-605 | 3.01E-01 | 93 | | 137 | 0.56 |
| hsa-miR-4767 | 3.01E-01 | 24 | | 70 | 1.55 |
| hsa-miR-194* | 3.04E-01 | 37 | | 19 | -0.94 |
| hsa-miR-4288 | 3.04E-01 | 15 | | 34 | 1.19 |
| hsa-miR-22* | 3.04E-01 | 48 | | 15 | -1.65 |
| hsa-miR-485-3p | 3.04E-01 | 79 | | 45 | -0.81 |
| hsa-miR-4723-3p | 3.05E-01 | 32 | | 21 | -0.63 |
| hsa-miR-3646 | 3.06E-01 | 39 | | 62 | 0.68 |
| hsa-miR-27a* | 3.08E-01 | 5 | | 21 | 2.04 |
| hsa-miR-3691-3p | 3.09E-01 | 13 | | 30 | 1.27 |
| hsa-miR-510 | 3.09E-01 | 36 | | 13 | -1.51 |
| hsa-miR-1303 | 3.10E-01 | 19 | | 33 | 0.78 |
| hsa-miR-648 | 3.12E-01 | 25 | | 34 | 0.45 |
| hsa-miR-193b* | 3.14E-01 | 85 | | 52 | -0.71 |
| hsa-miR-550a* | 3.14E-01 | 54 | | 26 | -1.06 |
| hsa-miR-1471 | 3.15E-01 | 20 | | 59 | 1.55 |
| hsa-miR-28-5p | 3.15E-01 | 71 | | 33 | -1.10 |
| hsa-miR-193a-3p | 3.15E-01 | 103 | | 58 | -0.83 |
| hsa-miR-370 | 3.17E-01 | 22 | | 42 | 0.95 |
| hsa-miR-378d | 3.17E-01 | 64 | | 33 | -0.94 |
| hsa-miR-4685-3p | 3.19E-01 | 43 | | 12 | -1.84 |
| hsa-miR-4769-3p | 3.20E-01 | 26 | | 68 | 1.37 |
| hsa-miR-4271 | 3.21E-01 | 50 | | 89 | 0.85 |
| hsa-miR-2278 | 3.22E-01 | 45 | | 30 | -0.59 |
| hsa-miR-296-3p | 3.22E-01 | 23 | | 40 | 0.81 |
| hsa-miR-3173-3p | 3.23E-01 | 18 | | 32 | 0.81 |
| hsa-miR-3150b-5p | 3.24E-01 | 31 | | 55 | 0.83 |
| hsa-miR-2467-3p | 3.24E-01 | 66 | | 25 | -1.37 |
| hsa-miR-1301 | 3.26E-01 | 23 | | 37 | 0.70 |
| hsa-miR-4749-3p | 3.27E-01 | 41 | | 52 | 0.36 |
| hsa-miR-191* | 3.27E-01 | 27 | | 65 | 1.28 |
| hsa-miR-4283 | 3.28E-01 | 24 | | 44 | 0.86 |
| hsa-miR-126 | 3.28E-01 | 57 | | 27 | -1.08 |
| hsa-miR-205 | 3.28E-01 | 31 | | 1 | -5.17 |
| hsa-miR-498 | 3.28E-01 | 34 | | 55 | 0.67 |
| hsa-miR-1202 | 3.31E-01 | 23 | | 44 | 0.91 |
| hsa-miR-4251 | 3.32E-01 | 30 | | 8 | -1.90 |
| hsa-miR-520d-5p | 3.32E-01 | 39 | | 28 | -0.50 |
| hsa-miR-4279 | 3.32E-01 | 25 | | 46 | 0.87 |
| hsa-miR-1291 | 3.34E-01 | 18 | | 33 | 0.90 |
| hsa-miR-30b* | 3.36E-01 | 28 | | 11 | -1.32 |
| hsa-miR-939 | 3.36E-01 | 60 | | 113 | 0.90 |
| hsa-miR-148b | 3.40E-01 | 69 | | 35 | -0.99 |
| hsa-miR-1973 | 3.40E-01 | 214 | | 282 | 0.40 |
| hsa-miR-4449 | 3.40E-01 | 36 | | 55 | 0.61 |
| hsa-miR-1237 | 3.41E-01 | 23 | | 66 | 1.51 |
| hsa-miR-3907 | 3.41E-01 | 28 | | 16 | -0.85 |
| hsa-miR-1225-3p | 3.41E-01 | 27 | | 43 | 0.68 |
| hsa-miR-627 | 3.41E-01 | 37 | | 13 | -1.51 |
| hsa-miR-877 | 3.43E-01 | 40 | | 74 | 0.90 |
| hsa-miR-3135 | 3.44E-01 | 16 | | 32 | 0.97 |
| hsa-miR-4297 | 3.44E-01 | 20 | | 38 | 0.96 |
| hsa-miR-4321 | 3.46E-01 | 16 | | 35 | 1.14 |
| hsa-miR-4512 | 3.47E-01 | 7 | | 29 | 2.08 |
| hsa-miR-4695-5p | 3.48E-01 | 79 | | 133 | 0.75 |
| hsa-miR-4728-3p | 3.48E-01 | 18 | | 40 | 1.20 |
| hsa-miR-4800-3p | 3.50E-01 | 213 | | 155 | -0.46 |
| hsa-miR-378e | 3.50E-01 | 68 | | 45 | -0.59 |
| hsa-miR-4725-5p | 3.51E-01 | 11 | | 52 | 2.25 |
| hsa-miR-1228 | 3.52E-01 | 32 | | 105 | 1.72 |
| hsa-miR-4472 | 3.54E-01 | 51 | | 111 | 1.12 |
| hsa-miR-3619-5p | 3.55E-01 | 15 | | 34 | 1.16 |
| hsa-miR-34a | 3.55E-01 | 62 | | 27 | -1.21 |
| hsa-miR-4436b-5p | 3.56E-01 | 31 | | 64 | 1.05 |
| hsa-miR-328 | 3.57E-01 | 11 | | 31 | 1.55 |
| hsa-miR-4638-5p | 3.58E-01 | 55 | | 40 | -0.45 |
| hsa-miR-4746-3p | 3.61E-01 | 26 | | 35 | 0.44 |
| hsa-miR-3173-5p | 3.61E-01 | 18 | | 30 | 0.73 |
| hsa-miR-518a-5p | 3.62E-01 | 9 | | 33 | 1.86 |
| hsa-miR-200a* | 3.62E-01 | 36 | | 14 | -1.37 |
| hsa-miR-887 | 3.69E-01 | 23 | | 58 | 1.35 |
| hsa-miR-3189-3p | 3.70E-01 | 19 | | 34 | 0.79 |
| hsa-miR-1208 | 3.72E-01 | 29 | | 54 | 0.91 |
| hsa-miR-4502 | 3.73E-01 | 43 | | 20 | -1.08 |
| hsa-miR-432 | 3.74E-01 | 44 | | 15 | -1.56 |
| hsa-miR-371b-3p | 3.74E-01 | 22 | | 36 | 0.70 |
| hsa-miR-326 | 3.74E-01 | 7 | | 28 | 2.04 |
| hsa-miR-196b | 3.75E-01 | 45 | | 31 | -0.52 |
| hsa-miR-92a-2* | 3.76E-01 | 15 | | 27 | 0.89 |
| hsa-miR-4784 | 3.76E-01 | 23 | | 34 | 0.57 |
| hsa-miR-3132 | 3.76E-01 | 26 | | 34 | 0.39 |
| hsa-miR-181d | 3.77E-01 | 96 | | 60 | -0.67 |
| hsa-miR-4445* | 3.79E-01 | 40 | | 9 | -2.14 |
| hsa-miR-630 | 3.79E-01 | 41 | | 49 | 0.27 |
| hsa-miR-4785 | 3.80E-01 | 26 | | 33 | 0.36 |
| hsa-miR-4422 | 3.81E-01 | 27 | | 19 | -0.52 |
| hsa-miR-4758-3p | 3.82E-01 | 44 | | 75 | 0.77 |
| hsa-miR-24-1* | 3.84E-01 | 27 | | 2 | -3.75 |
| hsa-miR-4526 | 3.85E-01 | 25 | | 39 | 0.66 |
| hsa-miR-514b-5p | 3.86E-01 | 73 | | 37 | -0.97 |
| hsa-miR-3622a-3p | 3.86E-01 | 3 | | 29 | 3.16 |
| hsa-miR-518a-3p | 3.87E-01 | 5 | | 26 | 2.33 |
| hsa-miR-3937 | 3.88E-01 | 46 | | 36 | -0.35 |
| hsa-miR-4317 | 3.88E-01 | 22 | | 49 | 1.15 |
| hsa-miR-4286 | 3.91E-01 | 163 | | 112 | -0.54 |
| hsa-miR-330-5p | 3.91E-01 | 9 | | 24 | 1.44 |
| hsa-miR-885-3p | 3.93E-01 | 17 | | 41 | 1.25 |
| hsa-miR-4467 | 3.95E-01 | 36 | | 77 | 1.09 |
| hsa-miR-1972 | 3.95E-01 | 133 | | 89 | -0.58 |
| hsa-miR-4440 | 3.96E-01 | 73 | | 49 | -0.58 |
| hsa-miR-4293 | 3.97E-01 | 18 | | 37 | 1.02 |
| hsa-miR-1470 | 3.98E-01 | 23 | | 64 | 1.45 |
| hsa-miR-4783-5p | 3.99E-01 | 34 | | 45 | 0.41 |
| hsa-miR-4287 | 4.00E-01 | 18 | | 32 | 0.84 |
| hsa-miR-125b-2* | 4.03E-01 | 55 | | 41 | -0.44 |
| hsa-miR-421 | 4.04E-01 | 37 | | 19 | -0.97 |
| hsa-miR-15a | 4.05E-01 | 78 | | 22 | -1.81 |
| hsa-miR-3943 | 4.05E-01 | 39 | | 22 | -0.81 |
| hsa-miR-637 | 4.06E-01 | 19 | | 44 | 1.21 |
| hsa-miR-4651 | 4.08E-01 | 95 | | 119 | 0.33 |
| hsa-miR-646 | 4.09E-01 | 18 | | 27 | 0.60 |
| hsa-miR-3691-5p | 4.13E-01 | 23 | | 31 | 0.42 |
| hsa-miR-4768-3p | 4.15E-01 | 15 | | 28 | 0.91 |
| hsa-let-7d* | 4.16E-01 | 38 | | 21 | -0.87 |
| hsa-miR-3064-3p | 4.17E-01 | 72 | | 38 | -0.93 |
| hsa-miR-215 | 4.20E-01 | 37 | | 27 | -0.46 |
| hsa-miR-3191 | 4.21E-01 | 19 | | 30 | 0.70 |
| hsa-miR-149 | 4.21E-01 | 22 | | 42 | 0.95 |
| hsa-miR-1246 | 4.23E-01 | 8,007 | | 6,755 | -0.25 |
| hsa-miR-4713-3p | 4.23E-01 | 27 | | 19 | -0.54 |
| hsa-miR-4300 | 4.23E-01 | 20 | | 34 | 0.80 |
| hsa-miR-935 | 4.24E-01 | 18 | | 35 | 0.94 |
| hsa-miR-3687 | 4.25E-01 | 9 | | 31 | 1.73 |
| hsa-miR-1269 | 4.28E-01 | 16 | | 38 | 1.24 |
| hsa-miR-4665-3p | 4.30E-01 | 24 | | 36 | 0.62 |
| hsa-miR-1305 | 4.33E-01 | 30 | | 39 | 0.38 |
| hsa-miR-3651 | 4.35E-01 | 23 | | 37 | 0.68 |
| hsa-miR-518d-3p | 4.39E-01 | 7 | | 22 | 1.54 |
| hsa-miR-891a | 4.41E-01 | 25 | | 17 | -0.62 |
| hsa-miR-187* | 4.41E-01 | 20 | | 48 | 1.26 |
| hsa-miR-3650 | 4.41E-01 | 13 | | 27 | 1.08 |
| hsa-miR-99b* | 4.42E-01 | 35 | | 17 | -1.07 |
| hsa-miR-1260 | 4.43E-01 | 48 | | 75 | 0.63 |
| hsa-miR-514b-3p | 4.43E-01 | 23 | | 4 | -2.53 |
| hsa-miR-34a* | 4.43E-01 | 15 | | 27 | 0.82 |
| hsa-miR-200b | 4.45E-01 | 26 | | 8 | -1.64 |
| hsa-miR-608 | 4.48E-01 | 24 | | 39 | 0.68 |
| hsa-miR-4436b-3p | 4.48E-01 | 304 | | 356 | 0.23 |
| hsa-miR-33b* | 4.52E-01 | 36 | | 52 | 0.51 |
| hsa-miR-3125 | 4.52E-01 | 65 | | 79 | 0.30 |
| hsa-miR-450b-3p | 4.54E-01 | 20 | | 2 | -3.12 |
| hsa-miR-4698 | 4.54E-01 | 106 | | 74 | -0.52 |
| hsa-miR-489 | 4.54E-01 | 42 | | 33 | -0.33 |
| hsa-miR-139-3p | 4.55E-01 | 30 | | 20 | -0.57 |
| hsa-miR-647 | 4.55E-01 | 36 | | 53 | 0.54 |
| hsa-miR-218 | 4.56E-01 | 27 | | 17 | -0.63 |
| hsa-miR-449c* | 4.57E-01 | 31 | | 16 | -0.94 |
| hsa-miR-1915* | 4.58E-01 | 24 | | 41 | 0.77 |
| hsa-let-7f-1* | 4.59E-01 | 11 | | 27 | 1.28 |
| hsa-miR-4302 | 4.61E-01 | 15 | | 30 | 0.99 |
| hsa-miR-4674 | 4.61E-01 | 43 | | 63 | 0.56 |
| hsa-miR-1231 | 4.61E-01 | 28 | | 53 | 0.94 |
| hsa-miR-148a* | 4.61E-01 | 9 | | 25 | 1.46 |
| hsa-miR-4428 | 4.62E-01 | 33 | | 51 | 0.64 |
| hsa-miR-4681 | 4.62E-01 | 24 | | 10 | -1.20 |
| hsa-miR-718 | 4.62E-01 | 45 | | 58 | 0.36 |
| hsa-miR-4483 | 4.63E-01 | 44 | | 55 | 0.31 |
| hsa-miR-555 | 4.64E-01 | 9 | | 22 | 1.30 |
| hsa-miR-3162-5p | 4.66E-01 | 62 | | 85 | 0.45 |
| hsa-miR-185* | 4.68E-01 | 29 | | 9 | -1.76 |
| hsa-miR-29c | 4.69E-01 | 54 | | 16 | -1.80 |
| hsa-miR-3921 | 4.70E-01 | 34 | | 9 | -1.92 |
| hsa-miR-4258 | 4.71E-01 | 12 | | 29 | 1.26 |
| hsa-miR-98 | 4.71E-01 | 163 | | 114 | -0.52 |
| hsa-miR-4514 | 4.74E-01 | 14 | | 31 | 1.18 |
| hsa-miR-612 | 4.75E-01 | 28 | | 21 | -0.40 |
| hsa-miR-4481 | 4.75E-01 | 235 | | 202 | -0.21 |
| hsa-miR-3174 | 4.75E-01 | 21 | | 34 | 0.73 |
| hsa-miR-19a* | 4.76E-01 | 26 | | 13 | -0.98 |
| hsa-miR-628-3p | 4.78E-01 | 28 | | 10 | -1.54 |
| hsa-miR-3193 | 4.80E-01 | 51 | | 44 | -0.22 |
| hsa-miR-3198 | 4.82E-01 | 26 | | 34 | 0.39 |
| hsa-miR-4489 | 4.82E-01 | 39 | | 54 | 0.46 |
| hsa-miR-4532 | 4.83E-01 | 970 | | 933 | -0.06 |
| hsa-miR-933 | 4.84E-01 | 57 | | 82 | 0.53 |
| hsa-miR-539 | 4.84E-01 | 38 | | 28 | -0.42 |
| hsa-miR-92b* | 4.84E-01 | 32 | | 44 | 0.47 |
| hsa-miR-4303 | 4.85E-01 | 32 | | 43 | 0.43 |
| hsa-miR-5095 | 4.85E-01 | 141 | | 105 | -0.43 |
| hsa-miR-4682 | 4.85E-01 | 25 | | 13 | -0.92 |
| hsa-miR-4310 | 4.87E-01 | 30 | | 42 | 0.52 |
| hsa-miR-4758-5p | 4.87E-01 | 50 | | 65 | 0.38 |
| hsa-miR-4646-5p | 4.87E-01 | 134 | | 144 | 0.10 |
| hsa-miR-1343 | 4.88E-01 | 24 | | 30 | 0.33 |
| hsa-miR-3138 | 4.90E-01 | 21 | | 29 | 0.49 |
| hsa-miR-676 | 4.90E-01 | 20 | | 7 | -1.48 |
| hsa-miR-4726-5p | 4.90E-01 | 26 | | 44 | 0.75 |
| hsa-miR-29b-1* | 4.91E-01 | 33 | | 14 | -1.29 |
| hsa-miR-3152-5p | 4.92E-01 | 218 | | 8 | -4.85 |
| hsa-miR-4772-5p | 4.93E-01 | 6 | | 19 | 1.58 |
| hsa-miR-29b | 4.94E-01 | 27 | | 12 | -1.20 |
| hsa-miR-3677-3p | 4.94E-01 | 26 | | 33 | 0.32 |
| hsa-miR-760 | 4.96E-01 | 39 | | 20 | -0.94 |
| hsa-miR-4669 | 4.98E-01 | 55 | | 79 | 0.53 |
| hsa-miR-377 | 4.98E-01 | 42 | | 31 | -0.45 |
| hsa-miR-550b | 4.99E-01 | 14 | | 23 | 0.77 |
| hsa-miR-4295 | 5.00E-01 | 11 | | 24 | 1.18 |
| hsa-miR-1229 | 5.00E-01 | 17 | | 33 | 0.94 |
| hsa-miR-4304 | 5.01E-01 | 103 | | 123 | 0.26 |
| hsa-miR-4763-5p | 5.02E-01 | 37 | | 18 | -1.07 |
| hsa-miR-147 | 5.03E-01 | 11 | | 24 | 1.20 |
| hsa-miR-509-3p | 5.03E-01 | 21 | | 7 | -1.52 |
| hsa-miR-92a-1* | 5.03E-01 | 26 | | 38 | 0.55 |
| hsa-miR-1296 | 5.04E-01 | 28 | | 45 | 0.67 |
| hsa-miR-4441 | 5.05E-01 | 29 | | 39 | 0.44 |
| hsa-miR-4754 | 5.06E-01 | 22 | | 33 | 0.61 |
| hsa-miR-3130-5p | 5.07E-01 | 41 | | 19 | -1.14 |
| hsa-miR-1295 | 5.09E-01 | 20 | | 34 | 0.74 |
| hsa-miR-1271 | 5.10E-01 | 21 | | 30 | 0.51 |
| hsa-miR-2113 | 5.13E-01 | 14 | | 25 | 0.89 |
| hsa-miR-4486 | 5.13E-01 | 87 | | 112 | 0.36 |
| hsa-miR-1226* | 5.14E-01 | 29 | | 18 | -0.72 |
| hsa-miR-154 | 5.15E-01 | 27 | | 17 | -0.69 |
| hsa-miR-324-3p | 5.15E-01 | 24 | | 38 | 0.68 |
| hsa-miR-1227 | 5.17E-01 | 32 | | 17 | -0.90 |
| hsa-miR-4305 | 5.22E-01 | 22 | | 33 | 0.58 |
| hsa-miR-1226 | 5.25E-01 | 21 | | 9 | -1.18 |
| hsa-miR-4315 | 5.26E-01 | 23 | | 16 | -0.54 |
| hsa-miR-4683 | 5.27E-01 | 31 | | 24 | -0.39 |
| hsa-miR-711 | 5.28E-01 | 36 | | 23 | -0.63 |
| hsa-miR-4664-5p | 5.28E-01 | 26 | | 35 | 0.45 |
| hsa-miR-4800-5p | 5.29E-01 | 275 | | 317 | 0.21 |
| hsa-miR-4455 | 5.29E-01 | 916 | | 1,143 | 0.32 |
| hsa-miR-219-2-3p | 5.30E-01 | 30 | | 22 | -0.45 |
| hsa-miR-3675-3p | 5.31E-01 | 37 | | 50 | 0.46 |
| hsa-miR-3622b-5p | 5.33E-01 | 21 | | 35 | 0.73 |
| hsa-miR-4728-5p | 5.33E-01 | 121 | | 112 | -0.10 |
| hsa-miR-21* | 5.33E-01 | 18 | | 30 | 0.79 |
| hsa-miR-4793-5p | 5.36E-01 | 23 | | 34 | 0.56 |
| hsa-miR-29c* | 5.36E-01 | 21 | | 16 | -0.35 |
| hsa-miR-3195 | 5.40E-01 | 111 | | 131 | 0.24 |
| hsa-miR-4285 | 5.40E-01 | 19 | | 30 | 0.71 |
| hsa-miR-4716-5p | 5.43E-01 | 41 | | 60 | 0.56 |
| hsa-miR-1538 | 5.48E-01 | 38 | | 24 | -0.67 |
| hsa-miR-2114* | 5.52E-01 | 15 | | 26 | 0.79 |
| hsa-miR-640 | 5.53E-01 | 19 | | 30 | 0.67 |
| hsa-miR-4755-3p | 5.53E-01 | 21 | | 27 | 0.40 |
| hsa-miR-4456 | 5.56E-01 | 20 | | 21 | 0.05 |
| hsa-miR-4498 | 5.59E-01 | 33 | | 44 | 0.41 |
| hsa-miR-652 | 5.60E-01 | 47 | | 38 | -0.30 |
| hsa-miR-4710 | 5.62E-01 | 193 | | 223 | 0.21 |
| hsa-miR-3156-5p | 5.65E-01 | 72 | | 50 | -0.53 |
| hsa-miR-3158-5p | 5.69E-01 | 38 | | 50 | 0.40 |
| hsa-miR-650 | 5.70E-01 | 29 | | 34 | 0.24 |
| hsa-miR-1914* | 5.72E-01 | 40 | | 44 | 0.14 |
| hsa-miR-1203 | 5.72E-01 | 34 | | 42 | 0.27 |
| hsa-miR-3914 | 5.72E-01 | 17 | | 7 | -1.25 |
| hsa-miR-1909 | 5.72E-01 | 22 | | 27 | 0.33 |
| hsa-miR-4646-3p | 5.73E-01 | 30 | | 20 | -0.58 |
| hsa-miR-4436a | 5.76E-01 | 28 | | 40 | 0.52 |
| hsa-miR-675* | 5.82E-01 | 31 | | 24 | -0.35 |
| hsa-miR-1182 | 5.82E-01 | 59 | | 70 | 0.26 |
| hsa-miR-2116 | 5.83E-01 | 28 | | 39 | 0.48 |
| hsa-miR-4451 | 5.84E-01 | 30 | | 24 | -0.35 |
| hsa-miR-551b* | 5.84E-01 | 33 | | 50 | 0.62 |
| hsa-miR-155 | 5.88E-01 | 38 | | 26 | -0.55 |
| hsa-miR-34c-3p | 5.90E-01 | 162 | | 137 | -0.24 |
| hsa-miR-374c | 5.92E-01 | 29 | | 22 | -0.40 |
| hsa-miR-409-3p | 5.92E-01 | 48 | | 60 | 0.33 |
| hsa-miR-1539 | 5.93E-01 | 29 | | 21 | -0.44 |
| hsa-miR-106b* | 6.00E-01 | 34 | | 31 | -0.13 |
| hsa-miR-509-5p | 6.02E-01 | 22 | | 13 | -0.76 |
| hsa-miR-4717-3p | 6.02E-01 | 73 | | 61 | -0.26 |
| hsa-miR-550a | 6.03E-01 | 41 | | 55 | 0.44 |
| hsa-miR-3923 | 6.05E-01 | 25 | | 11 | -1.14 |
| hsa-miR-1273c | 6.06E-01 | 47 | | 60 | 0.35 |
| hsa-miR-4685-5p | 6.07E-01 | 57 | | 72 | 0.34 |
| hsa-miR-4793-3p | 6.07E-01 | 15 | | 28 | 0.93 |
| hsa-miR-378c | 6.08E-01 | 16 | | 26 | 0.73 |
| hsa-miR-4676-3p | 6.10E-01 | 26 | | 19 | -0.44 |
| hsa-miR-4301 | 6.13E-01 | 27 | | 36 | 0.43 |
| hsa-miR-1286 | 6.13E-01 | 17 | | 24 | 0.53 |
| hsa-miR-4713-5p | 6.14E-01 | 24 | | 34 | 0.48 |
| hsa-miR-3151 | 6.15E-01 | 32 | | 41 | 0.37 |
| hsa-miR-4284 | 6.17E-01 | 187 | | 168 | -0.15 |
| hsa-miR-1180 | 6.19E-01 | 154 | | 144 | -0.10 |
| hsa-miR-4253 | 6.22E-01 | 43 | | 51 | 0.27 |
| hsa-miR-214 | 6.23E-01 | 55 | | 45 | -0.29 |
| hsa-miR-3944-3p | 6.24E-01 | 32 | | 25 | -0.35 |
| hsa-miR-4707-3p | 6.26E-01 | 36 | | 52 | 0.54 |
| hsa-let-7e | 6.28E-01 | 593 | | 519 | -0.19 |
| hsa-miR-2110 | 6.29E-01 | 21 | | 29 | 0.48 |
| hsa-miR-378* | 6.30E-01 | 29 | | 22 | -0.37 |
| hsa-miR-378b | 6.32E-01 | 57 | | 70 | 0.28 |
| hsa-miR-93* | 6.34E-01 | 32 | | 44 | 0.43 |
| hsa-miR-424* | 6.37E-01 | 39 | | 25 | -0.62 |
| hsa-miR-3917 | 6.41E-01 | 22 | | 32 | 0.50 |
| hsa-miR-181a-2* | 6.43E-01 | 28 | | 23 | -0.28 |
| hsa-miR-4501 | 6.47E-01 | 20 | | 10 | -0.97 |
| hsa-miR-1262 | 6.49E-01 | 20 | | 18 | -0.13 |
| hsa-miR-563 | 6.49E-01 | 28 | | 23 | -0.25 |
| hsa-miR-15a* | 6.50E-01 | 26 | | 20 | -0.35 |
| hsa-miR-675 | 6.50E-01 | 29 | | 24 | -0.27 |
| hsa-miR-3647-3p | 6.52E-01 | 19 | | 28 | 0.62 |
| hsa-miR-4749-5p | 6.55E-01 | 72 | | 75 | 0.07 |
| hsa-miR-3170 | 6.56E-01 | 37 | | 31 | -0.26 |
| hsa-miR-3919 | 6.58E-01 | 18 | | 10 | -0.84 |
| hsa-miR-574-5p | 6.61E-01 | 4,169 | | 4,362 | 0.07 |
| hsa-miR-4714-5p | 6.63E-01 | 24 | | 17 | -0.52 |
| hsa-miR-3922-5p | 6.69E-01 | 19 | | 25 | 0.40 |
| hsa-miR-135b* | 6.70E-01 | 28 | | 33 | 0.23 |
| hsa-miR-362-5p | 6.78E-01 | 33 | | 25 | -0.37 |
| hsa-miR-4701-5p | 6.84E-01 | 62 | | 52 | -0.26 |
| hsa-miR-615-5p | 6.90E-01 | 36 | | 30 | -0.29 |
| hsa-miR-1306 | 6.96E-01 | 114 | | 125 | 0.12 |
| hsa-miR-4665-5p | 6.97E-01 | 27 | | 33 | 0.31 |
| hsa-miR-4756-5p | 6.97E-01 | 45 | | 38 | -0.26 |
| hsa-miR-508-5p | 7.00E-01 | 29 | | 19 | -0.63 |
| hsa-miR-518e | 7.00E-01 | 14 | | 18 | 0.39 |
| hsa-miR-17* | 7.01E-01 | 31 | | 21 | -0.54 |
| hsa-miR-19b-1* | 7.01E-01 | 31 | | 27 | -0.20 |
| hsa-miR-4482 | 7.01E-01 | 28 | | 22 | -0.30 |
| hsa-miR-198 | 7.02E-01 | 40 | | 47 | 0.23 |
| hsa-miR-764 | 7.06E-01 | 43 | | 48 | 0.16 |
| hsa-miR-376c | 7.08E-01 | 11 | | 21 | 0.89 |
| hsa-miR-4687-5p | 7.09E-01 | 19 | | 20 | 0.03 |
| hsa-miR-4721 | 7.09E-01 | 38 | | 32 | -0.23 |
| hsa-miR-4446-3p | 7.12E-01 | 21 | | 25 | 0.22 |
| hsa-miR-2277-5p | 7.12E-01 | 25 | | 29 | 0.21 |
| hsa-miR-1269b | 7.14E-01 | 20 | | 30 | 0.58 |
| hsa-miR-378f | 7.15E-01 | 20 | | 29 | 0.53 |
| hsa-miR-4700-3p | 7.15E-01 | 26 | | 28 | 0.08 |
| hsa-miR-1909* | 7.16E-01 | 24 | | 29 | 0.25 |
| hsa-miR-339-3p | 7.17E-01 | 22 | | 25 | 0.19 |
| hsa-miR-518b | 7.23E-01 | 15 | | 24 | 0.67 |
| hsa-miR-1292 | 7.23E-01 | 28 | | 34 | 0.29 |
| hsa-miR-3945 | 7.25E-01 | 30 | | 26 | -0.16 |
| hsa-miR-4491 | 7.26E-01 | 27 | | 30 | 0.17 |
| hsa-miR-1263 | 7.28E-01 | 24 | | 21 | -0.16 |
| hsa-miR-1322 | 7.28E-01 | 18 | | 24 | 0.40 |
| hsa-miR-4672 | 7.29E-01 | 38 | | 33 | -0.22 |
| hsa-miR-466 | 7.32E-01 | 1,632 | | 1,796 | 0.14 |
| hsa-miR-1270 | 7.36E-01 | 16 | | 24 | 0.62 |
| hsa-miR-541* | 7.37E-01 | 20 | | 25 | 0.30 |
| hsa-miR-3918 | 7.38E-01 | 38 | | 44 | 0.22 |
| hsa-miR-3154 | 7.41E-01 | 45 | | 49 | 0.12 |
| hsa-miR-4267 | 7.42E-01 | 50 | | 47 | -0.08 |
| hsa-miR-4706 | 7.46E-01 | 29 | | 26 | -0.14 |
| hsa-miR-19b | 7.56E-01 | 385 | | 425 | 0.14 |
| hsa-miR-411* | 7.57E-01 | 23 | | 12 | -0.94 |
| hsa-miR-4289 | 7.57E-01 | 25 | | 31 | 0.31 |
| hsa-miR-641 | 7.59E-01 | 38 | | 36 | -0.11 |
| hsa-miR-4313 | 7.61E-01 | 29 | | 34 | 0.20 |
| hsa-miR-513a-5p | 7.63E-01 | 52 | | 45 | -0.22 |
| hsa-miR-602 | 7.63E-01 | 30 | | 37 | 0.31 |
| hsa-miR-4786-3p | 7.64E-01 | 26 | | 30 | 0.22 |
| hsa-miR-300 | 7.67E-01 | 34 | | 34 | 0.01 |
| hsa-miR-345 | 7.71E-01 | 43 | | 49 | 0.20 |
| hsa-miR-572 | 7.71E-01 | 41 | | 39 | -0.07 |
| hsa-miR-4667-3p | 7.72E-01 | 36 | | 42 | 0.23 |
| hsa-miR-1266 | 7.73E-01 | 27 | | 40 | 0.54 |
| hsa-miR-4500 | 7.76E-01 | 31 | | 33 | 0.06 |
| hsa-miR-3591-3p | 7.83E-01 | 590 | | 634 | 0.11 |
| hsa-miR-4450 | 7.83E-01 | 29 | | 23 | -0.30 |
| hsa-miR-4323 | 7.86E-01 | 24 | | 27 | 0.18 |
| hsa-miR-564 | 7.90E-01 | 58 | | 56 | -0.04 |
| hsa-miR-3978 | 7.93E-01 | 30 | | 23 | -0.40 |
| hsa-miR-3934 | 7.99E-01 | 21 | | 15 | -0.49 |
| hsa-miR-4439 | 8.00E-01 | 31 | | 38 | 0.28 |
| hsa-miR-554 | 8.00E-01 | 31 | | 30 | -0.02 |
| hsa-miR-423-3p | 8.03E-01 | 38 | | 28 | -0.46 |
| hsa-miR-3925-5p | 8.06E-01 | 28 | | 29 | 0.09 |
| hsa-miR-4689 | 8.06E-01 | 74 | | 76 | 0.04 |
| hsa-miR-3148 | 8.14E-01 | 146 | | 140 | -0.05 |
| hsa-miR-129-5p | 8.14E-01 | 34 | | 36 | 0.07 |
| hsa-miR-4673 | 8.15E-01 | 50 | | 57 | 0.17 |
| hsa-miR-3911 | 8.17E-01 | 24 | | 21 | -0.18 |
| hsa-miR-1273d | 8.20E-01 | 17 | | 22 | 0.35 |
| hsa-miR-566 | 8.21E-01 | 23 | | 27 | 0.23 |
| hsa-miR-4458 | 8.26E-01 | 20 | | 19 | -0.07 |
| hsa-miR-449b* | 8.31E-01 | 36 | | 30 | -0.26 |
| hsa-miR-302c* | 8.31E-01 | 15 | | 19 | 0.36 |
| hsa-miR-4430 | 8.35E-01 | 47 | | 52 | 0.14 |
| hsa-miR-422a | 8.39E-01 | 39 | | 42 | 0.09 |
| hsa-miR-4296 | 8.40E-01 | 31 | | 37 | 0.27 |
| hsa-miR-4462 | 8.40E-01 | 118 | | 125 | 0.09 |
| hsa-miR-4708-5p | 8.42E-01 | 24 | | 30 | 0.35 |
| hsa-miR-4432 | 8.42E-01 | 17 | | 11 | -0.69 |
| hsa-let-7b* | 8.53E-01 | 24 | | 26 | 0.15 |
| hsa-miR-193a-5p | 8.59E-01 | 73 | | 71 | -0.04 |
| hsa-miR-4448 | 8.60E-01 | 104 | | 101 | -0.04 |
| hsa-miR-4701-3p | 8.70E-01 | 228 | | 218 | -0.06 |
| hsa-miR-212 | 8.73E-01 | 28 | | 27 | -0.08 |
| hsa-miR-378i | 8.80E-01 | 28 | | 28 | 0.01 |
| hsa-miR-4675 | 8.84E-01 | 27 | | 36 | 0.39 |
| hsa-miR-3936 | 8.84E-01 | 28 | | 21 | -0.45 |
| hsa-miR-2277-3p | 8.86E-01 | 30 | | 32 | 0.08 |
| hsa-miR-4700-5p | 8.89E-01 | 57 | | 53 | -0.12 |
| hsa-miR-610 | 8.90E-01 | 30 | | 28 | -0.07 |
| hsa-miR-425* | 8.91E-01 | 40 | | 35 | -0.19 |
| hsa-miR-4485 | 8.91E-01 | 256 | | 267 | 0.06 |
| hsa-miR-4711-3p | 8.92E-01 | 19 | | 23 | 0.26 |
| hsa-miR-1224-3p | 8.95E-01 | 24 | | 18 | -0.43 |
| hsa-miR-4487 | 9.02E-01 | 38 | | 37 | -0.02 |
| hsa-miR-3064-5p | 9.02E-01 | 22 | | 22 | -0.01 |
| hsa-miR-509-3-5p | 9.04E-01 | 24 | | 19 | -0.38 |
| hsa-miR-1265 | 9.05E-01 | 14 | | 20 | 0.54 |
| hsa-miR-346 | 9.06E-01 | 25 | | 26 | 0.06 |
| hsa-miR-941 | 9.08E-01 | 22 | | 16 | -0.47 |
| hsa-miR-1273e | 9.10E-01 | 24 | | 25 | 0.06 |
| hsa-miR-4695-3p | 9.13E-01 | 23 | | 20 | -0.21 |
| hsa-miR-105 | 9.16E-01 | 21 | | 19 | -0.13 |
| hsa-miR-219-1-3p | 9.18E-01 | 26 | | 28 | 0.11 |
| hsa-miR-3972 | 9.19E-01 | 28 | | 34 | 0.27 |
| hsa-miR-1911* | 9.22E-01 | 23 | | 21 | -0.07 |
| hsa-miR-2276 | 9.22E-01 | 31 | | 30 | -0.03 |
| hsa-miR-1910 | 9.24E-01 | 32 | | 31 | -0.03 |
| hsa-miR-4709-3p | 9.34E-01 | 36 | | 37 | 0.03 |
| hsa-miR-3682-3p | 9.36E-01 | 129 | | 145 | 0.17 |
| hsa-miR-3926 | 9.38E-01 | 30 | | 28 | -0.11 |
| hsa-miR-1914 | 9.39E-01 | 28 | | 27 | -0.05 |
| hsa-miR-195* | 9.40E-01 | 26 | | 27 | 0.08 |
| hsa-miR-4715-5p | 9.43E-01 | 29 | | 25 | -0.24 |
| hsa-miR-4664-3p | 9.44E-01 | 30 | | 30 | -0.01 |
| hsa-miR-611 | 9.44E-01 | 24 | | 24 | -0.02 |
| hsa-miR-378g | 9.45E-01 | 31 | | 35 | 0.14 |
| hsa-miR-4753-5p | 9.46E-01 | 46 | | 47 | 0.02 |
| hsa-miR-4722-3p | 9.51E-01 | 32 | | 30 | -0.09 |
| hsa-miR-4461 | 9.51E-01 | 25 | | 14 | -0.88 |
| hsa-miR-181c* | 9.56E-01 | 269 | | 236 | -0.19 |
| hsa-miR-501-3p | 9.61E-01 | 31 | | 31 | -0.03 |
| hsa-miR-1293 | 9.63E-01 | 29 | | 24 | -0.31 |
| hsa-miR-4468 | 9.66E-01 | 32 | | 28 | -0.17 |
| hsa-miR-936 | 9.67E-01 | 62 | | 64 | 0.04 |
| hsa-miR-4708-3p | 9.71E-01 | 40 | | 41 | 0.05 |
| hsa-miR-2115* | 9.73E-01 | 44 | | 44 | -0.01 |
| hsa-miR-4718 | 9.76E-01 | 20 | | 16 | -0.34 |
| hsa-miR-1976 | 9.77E-01 | 33 | | 35 | 0.08 |
| hsa-miR-3661 | 9.78E-01 | 31 | | 33 | 0.07 |
| hsa-miR-28-3p | 9.78E-01 | 23 | | 25 | 0.11 |
| hsa-miR-4479 | 9.79E-01 | 33 | | 30 | -0.14 |
| hsa-miR-4688 | 9.79E-01 | 28 | | 25 | -0.14 |
| hsa-miR-133b | 9.84E-01 | 24 | | 25 | 0.07 |
| hsa-miR-4476 | 9.86E-01 | 31 | | 20 | -0.60 |
| hsa-miR-4717-5p | 9.87E-01 | 31 | | 27 | -0.18 |
| hsa-miR-671-3p | 9.87E-01 | 31 | | 31 | -0.03 |
| hsa-miR-4719 | 9.90E-01 | 24 | | 21 | -0.20 |
| hsa-miR-4435 | 9.90E-01 | 27 | | 27 | -0.02 |
| hsa-miR-1323 | 9.91E-01 | 26 | | 27 | 0.06 |
| hsa-miR-3150b-3p | 9.93E-01 | 32 | | 30 | -0.06 |
| hsa-miR-1273f | 9.93E-01 | 35 | | 36 | 0.05 |
| hsa-miR-4506 | 9.94E-01 | 50 | | 49 | -0.05 |
| hsa-miR-1261 | 9.94E-01 | 18 | | 24 | 0.37 |
| hsa-miR-223 | 9.94E-01 | 31 | | 32 | 0.03 |
| hsa-miR-3677-5p | 9.97E-01 | 31 | | 30 | -0.03 |
| hsa-miR-511 | 9.99E-01 | 17 | | 8 | -1.09 |

1. **Fold difference (log2 (Ratio of miR-138/control)) >0.30 (up-regulated) or <-0.30 (down-regulated), and p-value < 0.1.**

### Supplementary Figure 1. Mascot Score Histogram.Ions score is -10*Log(P), where P is the probability that the observed match is a random event. Individual ions scores > 38 indicate identity or extensive homology (p<0.05). Protein scores are derived from ions scores as a non-probabilistic basis for ranking protein hits.


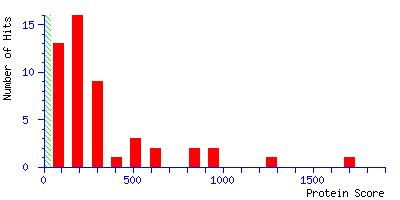


**Supplementary Figure 2**. miR-138 does not directly target Exportin-5 and Dicer.(**A**) Western blot analyses show that miR-138 downregulates Exportin-5 protein and reduces Dicer protein expression. (**B**) qRT-PCR assays were performed to examine the effects of miR-138 mimic transfection on endogenous RMND5A, Exportin-5 and Dicer gene transcription. (**C**) Dual luciferase reporter assays were performed to test the interaction of miR-138 with four fragments of DICER 3’UTR (pGL3-D1, pGL3-D2, pGL3-D3, and pGL3-D4) and full-length XPO5 3’UTR. Data are representative of three independent experiments (mean and s.d.). ^＊^*P <* 0.05 versus control.


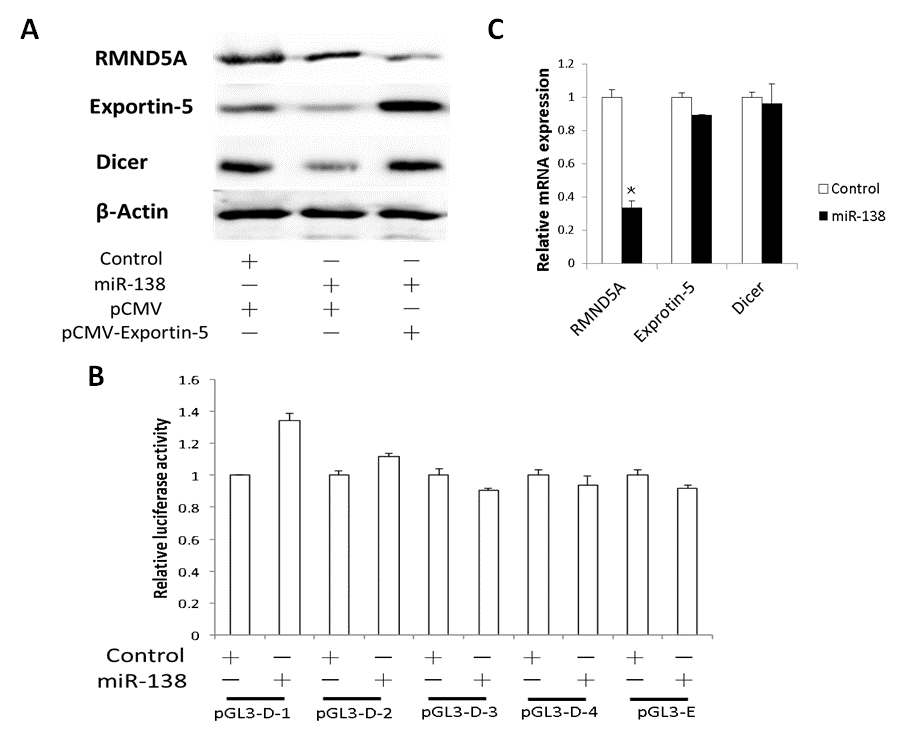


**Supplementary Figure 3.** Dicer and Exportin-5 expression levels have little effect on HeLa cell migration. (**A**) Quantitative RT-PCR assays were performed to examine the effects of Dicer siRNA and Exportin-5 siRNA transfection on endogenous DICER and XPO5 gene transcription. (**B**) Phase micrographs of migrated HeLa cells stained with DAPI. HeLa cells were transiently transfected with Dicer siRNA, Exportin-5 siRNA, pCMV-Dicer, and pCMV-Exprotin-5, and the motility of the transfected cells was evaluated by transwell migration assays. (**C**) Relative cell migration was determined by the number of DAPI-stained cells that migrated to the underside of the ﬁlter, normalized to the number of cells transfected with control siRNA or control plasmid. Cell migration is expressed as a percentage of that observed in the control and data are the means±s.d. of three independent experiments. ^＊^*P <* 0.05 versus control.


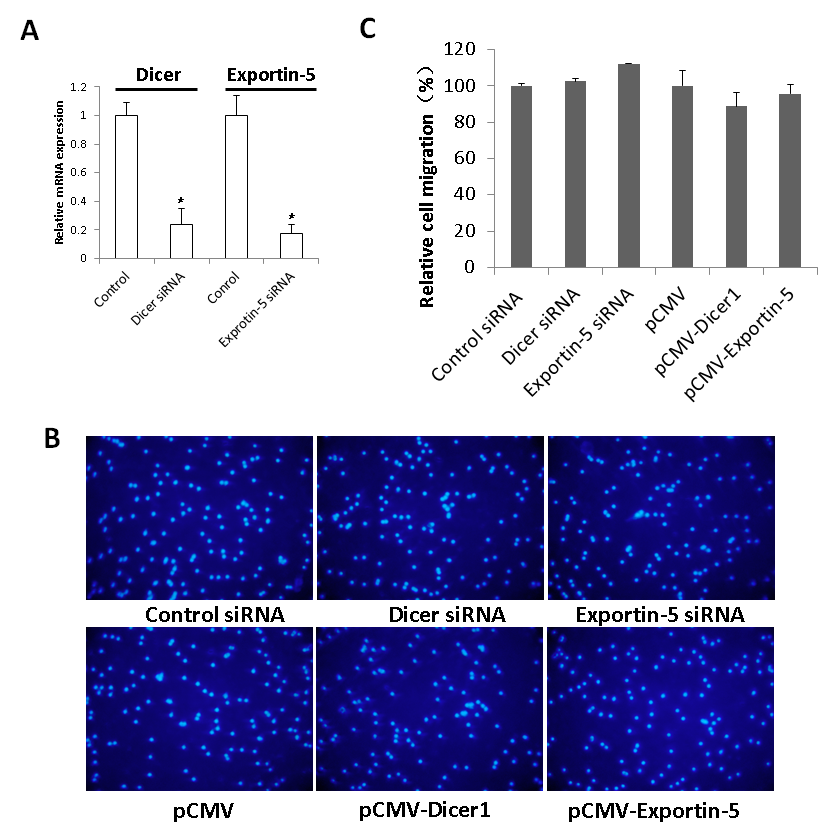


**Supplementary Figure 4.** Expression Analysis of RMND5a in miR-138 mimic and Control. The plot shows expression of RMND5A in airway epithelia cells (Calu-3) with miR-138 mimic treatment compared to that in control (P=0.000115). The microarray data is from GEO (http://www.ncbi.nlm.nih.gov/geo) and Normalized by RMA.

**
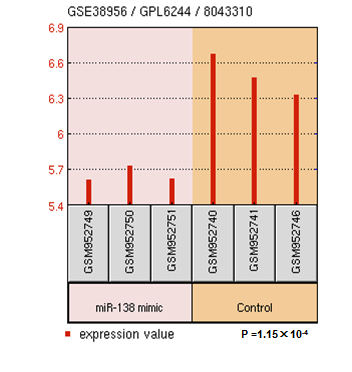
**
